# Establishment and validation of a carbohydrate metabolism-related gene signature for prognostic model and immune response in acute myeloid leukemia

**DOI:** 10.3389/fimmu.2022.1038570

**Published:** 2022-12-05

**Authors:** You Yang, Yan Yang, Jing Liu, Yan Zeng, Qulian Guo, Jing Guo, Ling Guo, Haiquan Lu, Wenjun Liu

**Affiliations:** ^1^ Department of Pediatrics (Children Hematological Oncology), Birth Defects and Childhood Hematological Oncology Laboratory, The Affiliated Hospital of Southwest Medical University, Sichuan Clinical Research Center for Birth Defects, Luzhou, Sichuan, China; ^2^ Department of Hematology, The Affiliated Hospital of Southwest Medical University. Luzhou, Sichuan, China; ^3^ The Second Hospital, Center for Reproductive Medicine, Advanced Medical Research Institute, and Key Laboratory for Experimental Teratology of the Ministry of Education, Cheeloo College of Medicine, Shandong University, Jinan, Shandong, China

**Keywords:** acute myeloid leukemia, carbohydrate metabolism, prognosis, immune response, therapeutic response

## Abstract

**Introduction:**

The heterogeneity of treatment response in acute myeloid leukemia (AML) patients poses great challenges for risk scoring and treatment stratification. Carbohydrate metabolism plays a crucial role in response to therapy in AML. In this multicohort study, we investigated whether carbohydrate metabolism related genes (CRGs) could improve prognostic classification and predict response of immunity and treatment in AML patients.

**Methods:**

Using univariate regression and LASSO-Cox stepwise regression analysis, we developed a CRG prognostic signature that consists of 10 genes. Stratified by the median risk score, patients were divided into high-risk group and low-risk group. Using TCGA and GEO public data cohorts and our cohort (1031 non-M3 patients in total), we demonstrated the consistency and accuracy of the CRG score on the predictive performance of AML survival.

**Results:**

The overall survival (OS) was significantly shorter in high-risk group. Differentially expressed genes (DEGs) were identified in the high-risk group compared to the low-risk group. GO and GSEA analysis showed that the DEGs were mainly involved in immune response signaling pathways. Analysis of tumor-infiltrating immune cells confirmed that the immune microenvironment was strongly suppressed in high-risk group. The results of potential drugs for risk groups showed that inhibitors of carbohydrate metabolism were effective.

**Discussion:**

The CRG signature was involved in immune response in AML. A novel risk model based on CRGs proposed in our study is promising prognostic classifications in AML, which may provide novel insights for developing accurate targeted cancer therapies.

## Introduction

Acute myeloid leukemia (AML) is a highly genetically, epigenetically, and clinically heterogeneous disease characterized by clonal expansion of undifferentiated myeloid precursors, resulting in impaired hematopoiesis and bone marrow failure (1, 2). AML is more common in middle-aged and older adults, with a median age at diagnosis of 68 years (3). Standard curative treatments for AML include chemotherapy alone and allogeneic stem cell transplantation in combination with chemotherapy (4). The long-term survival probability ranges from approximately 35% to 40% for AML patients under the age of 60 and from 5% to 15% for patients over the age of 60. Relapse is common in majority of elderly AML patients (5, 6). Occurrence, development and prognosis of AML is complicated and need to be continuously explored. The long-term survival and continuous proliferation of cancer cells are the prerequisites for the development of cancers. To meet the growing bioenergetic and biosynthetic demands for survival and proliferation, cancer cells autonomously regulate fluxes of metabolites through various metabolic pathways, including fatty acid metabolism, amino acid metabolism, and carbohydrate metabolism (7–9).

Carbohydrate is one of the most important biomolecules in living organisms. In addition to providing biological energy for physiological activities, carbohydrate metabolism also produce abundant metabolites for biosynthesis (10). Based on their structures, carbohydrates are divided into simple sugars, complex carbohydrates and glycoconjugates, which exert different biological functions through different metabolic pathways (10). Simple sugars and complex carbohydrates can enter glycolysis for energy metabolism (10, 11), while glycoconjugates, such as glycolipids and glycoproteins, are involved in many complicated biological processes (12). Numerous research has reported that carbohydrate metabolic activities and products are involved in critical process in cancers, including tumor initiation, angiogenesis, angiogenesis, metastasis to distant organs, invasion, and therapeutic resistance (10, 13–16). In addition, carbohydrate metabolism also plays a key role in influencing and predicting tumor prognosis (17). It has been reported that carbohydrate metabolism is significantly enhanced in AML cells, and glycolysis inhibitor R-2-hydroxyglutarate has anti-tumor activity in AML (18). MCL-1 inhibitors resensitizes AML to BCL-2 inhibition by regulating leukemia cell bioenergetics and carbohydrate metabolism, including the TCA cycle, glycolysis and pentose phosphate pathway and modulating cell adhesion proteins and leukemia-stromal interactions (19). Inhibition of oxidative phosphorylation (OXPHOS) causes energy deprivation and impaired nucleotide biosynthesis, which ultimately leads to inhibition of AML cell proliferation, induction of apoptosis, and prolonged survival in mouse models (20). It has been reported that inhibition of Nrf2-mediated glucose metabolism sensitizes AML to Ara-C (21). Previous work of carbohydrate metabolism in AML is mainly focused on preclinical studies. However, the relationship between carbohydrate metabolism and immunity, gene mutation, and prognosis in AML has not yet been clinically reported.

**Figure 1:**
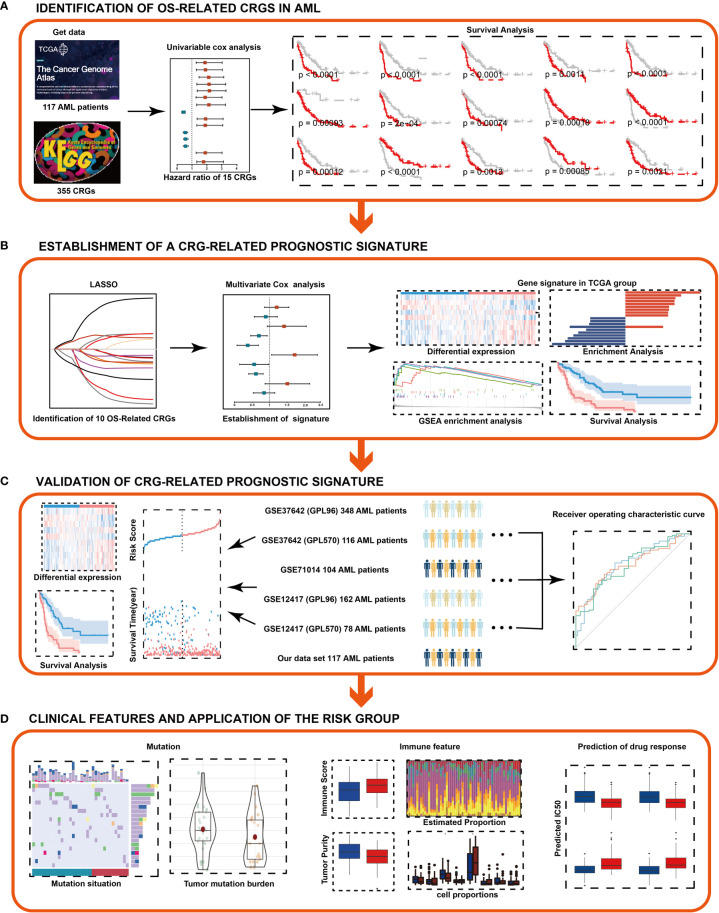
Flowchart of the study. **(A)** Identification of OS-related CRGs in AML. **(B)** Combined approaches were used to establish a robust CRG signature for prognosis. **(C)** Prognostic value of the CRG signature was validated in different cohorts. **(D)** Clinical features and application of the risk group. LASSO, least absolute shrinkage and selection operator.

In the present study, we analyzed samples from 1031 AML patients for the relationship of carbohydrate metabolism-related genes with prognosis and drug responses. To systematically describe our study, the flowchart was presented (**Figure 1**). By utilizing the AML training cohort from the Cancer Genome Atlas (TCGA) database, we performed the least absolute shrinkage and selection operator (LASSO) Cox analysis (22) and constructed a carbohydrate metabolism-related gene (CRG) signature related with prognosis. Prognostic value of the CRG signature was internally validated in the TCGA training set, and was further externally validated in the GEO testing cohort and our own cohort. Based on the CRG signature, we explored characteristics of mutation and immune cells by CIBERSORT (23) and Maftools package (24). Furthermore, we evaluated the ability of the CRG signature to predict patient response to chemotherapeutic drugs by OncoPredict (25).

## Material and method

### Publicly available datasets and preprocessing

The RNA sequencing profiles, single nucleotide polymorphism (SNP) profiles and detailed clinical data of AML datasets were downloaded from public databases. Raw microarray datasets of GSE37642 (GPL 570, n = 140), GSE37642 (GPL 96, n = 422), GSE71014 (GPL 10558, n = 104), GSE12417 (GPL 96, n = 162) and GSE12417 (GPL 570, n = 78) were downloaded from the GEO (https://www.ncbi.nlm.nih.gov/geo/) and normalized by normalizeBetweenArrays function using the limma package (26) between array. The AML RNA-seq dataset was downloaded from The Cancer Genome Atlas (TCGA) database (https://portal.gdc.cancer.gov/repository). 151 AML patients were included in the TCGA database. M3-AML is a relatively well-characterized subtype of AML. The etiology, molecular mechanisms, and treatment of M3-AML have been comprehensively studied. Therefore, this type of AML was excluded from the clinical specimens of this study. Non-M3 AML patients were finally selected to further analysis. 925 AML patients were enrolled (**Figure 1**). The available clinical information of AML patients was listed in **Supplementary Table S1-6**. The pbmc3k dataset was downloaded from the 10X Genomics website (https://satijalab.org/seurat/articles/pbmc3k_tutorial.html).

### Our cohort

AML patients were diagnosed based on WHO 2008 criteria and classified according to FAB classification. The treatment of AML patients is mainly based on the Chinese guidelines for diagnosis and treatment of adult AML (not APL) (2017) (27) at the time of admission. ELN Criteria were used for prognostication and of AML patients. Briefly, 106 newly diagnosed AML patients (non-M3 subtype) were finally selected in Affiliated Hospital of Southwest Medical University from January 2019 to June 2022. The detailed clinical information was obtained. The study approved by the Affiliated Hospital of Southwest Medical University was conducted in accordance with the Declaration of Helsinki. The available clinical information and critical gene alterations of AML samples in our cohort were listed in **Supplementary Table S7**.

### Acquisition of CRGs

A total of 355 CRGs were derived from the Kyoto Encyclopedia of Genes and Genomes (KEGG) pathway database (https://www.genome.jp/kegg/pathway.html#global). They were available **Supplementary Table S9**. The KEGG pathway database constitutes the reference knowledge base for understanding higher-level systemic functions of the cell and the organism, including metabolism, other cellular processes, organismal functions and human diseases (28).

### Identification of OS-related CRGs

To explore the potential prognostic significance of these CRGs in AML patients, the TCGA cohort (n = 117) was applied as a training set to identify OS-related CRGs with P-values less than 0.01 by univariate Cox proportional hazards regression analysis.

### Construction and validation of CRG prognostic signature for AML patients

Based Cox regression, the least absolute shrinkage and selection operator (LASSO) (29) was used to get the most significant features within the OS-related CRGs. Next, based on the Akaike information criterion, a multivariate Cox proportional hazards regression was performed on these candidates with the stepwise selection of variables (30). Risk score for final prognostic features were calculated as follows:


$$Risk\;score=\sum_{i}^{n}Coefi\times A_{i}$$

where Coef is the regression coefficient, “i” represents the CRG that composed of the CRG signature, “A” represents the relative expression value of the individual CRG, and “n” represents the number of genes in the signature. Patients were divided into high-risk and low-risk groups according to the median risk score as a cutoff value. Kaplan-Meier analysis and log-rank test were used to assess differences in the OS of patients. The predictive capacity of the CRG signature was evaluated by the time-dependent receiver operating characteristic (ROC) curve (31).

To test the predictive accuracy of the CRG signature, three external AML cohorts — GSE37642 (GPL 570, n=140), GSE37642 (GPL 96, n=422), and our cohort (n = 106) — were used as validation sets. Based on the CRG signature, the risk score of each patient was calculated, and Kaplan–Meier curve was used to reflect its survival performance.

### Identification and enrichment analysis of differentially expressed genes

The DEseq2 package was applied to identify the differentially expressed genes (DEGs) between the high- and low-risk groups (32).Heatmap for the DEGs was generated *via* ‘pheatmap’ package (33).

Though the clusterProfiler package, the gene o ntology (GO) functional enrichment analysis and GSEA analysis were utilized to better understand the functions of the DEGs in AML (34, 35).

### Development of carbohydrate metabolism clinicopathologic nomogram

To predict the OS of each AML patient, a carbohydrate metabolism nomogram that incorporated the CRG signature into the clinicopathologic parameters available in the training set was conducted through the rms package (36). The predictive discrimination of the CRG signature for AML patients was assessed by calibration curve (37).

### AML-immune microenvironment landscape and potential implications defined by the CRG signature

After selecting the 117 training samples, we extracted the transcriptome data and then calculated the immune purity and immune infiltration (based on the ImmuneScore, StromalScore, and ESTIMATEScore of the expression matrix using the “estimate” R package (38).

The proportions of 22 immune cell types (i.e., TICs) in each of the 117 AML samples (with immune infiltration scores) were calculated using the CIBERSORT algorithm and visualized using bar charts (23). Samples with P< 0.05 were chosen for further analysis. The proportions were then compared between tumor tissues with low- or high-risk group using Wilcoxon rank sum test, and Pearson’s correlation was assessed between the proportions. The results were presented in histograms, boxplots and heatmap, respectively, using the “ggplot”, “corrplot”, and “corrplot” R packages.

### Prediction of clinical chemotherapeutic response

OncoPredict is an R package for predicting the drug response (25), by which the associations of risk groups with the sensitivities to the commonly utilized chemotherapy and molecular targeted drugs were investigated. The results were presented in boxplots, using the “ggplot2” R packages.

### Gene expression levels were obtained by quantitative real-time PCR

RZ solution (Transgen, China) was used to extract total RNA from peripheral blood mononuclear cells (PBMCs) of 106 newly diagnosed AML patients in our hospital. cDNA was synthesized using a TranScript All-in-One First-Strand cDNA Synthesis SuperMix for qPCR kit (Transgen, China), and TranStart Tip Green Qpcr SuperMix (Transgen, China) was used to assay the HOXA5 mRNA levels. The amplification proceeded as follows: 94°C, 30 seconds, 1 cycle; 94°C, 5 seconds, 60°C, 30 seconds, 40 cycles. The primers were listed in **Supplementary Table S8**. The 2−ΔΔ CT method was used to calculate relative mRNA expression.

## Statistical analysis

Statistical analyses were performed using R software version 4.2.0 (https://www.r-project.org/). OS were estimated according to the Kaplan-Meier method and log-rank test were performed. The code had been submitted to github (https://github.com/jmzeng1314/TCGA_AML_Glycolysis). Statistical differences between risk groups were determined using χ^2^, Mann-Whitney, or Fisher’s exact tests when appropriate. The comparison of indicated two groups was performed by Student’s t-test (two tailed, unpaired): *p< 0.05; **p< 0.01; ***p< 0.001; ns, not significant.

## Results

### Identification of survival-related CRGs in AML

**Figure 2:**
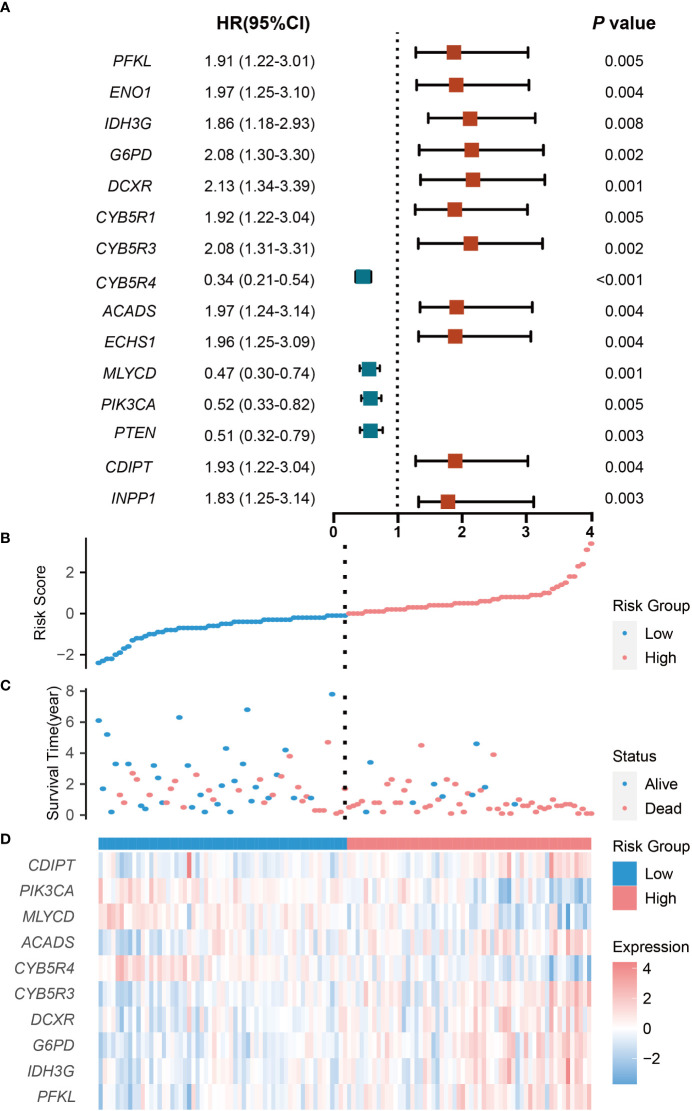
Prognostic analysis of the CRG signature in the training set. **(A)** The hazard ratio of CRGs associated with OS. **(B)** Risk Groups based on patients’ risk scores. **(C)** Patients’ survival status along with their risk scores. **(D)** The expressions of the 10 model CRGs in the high- and low-risk groups. CRGs with P values less than 0.01 were selected. HR indicates hazard ratio; CI indicates confidence interval.

Gene expression profiles of 117 non-M3 AML samples in TCGA dataset were selected and defined as the training set. We obtained 355 CRGs from the KEGG dataset and performed univariate Cox proportional hazards regression analysis to explore the potential prognostic value of each CRG. We found 15 CRGs that were significantly associated with OS (**Figure 2A**). Among these 15 genes, four genes (CYB5R4, MLYCD, PIK3CA and PTEN) were protective factors, and the rest 11 genes were risk factors (**Figure 2A**). More importantly, survival analysis showed that high expression of these four genes was significantly correlated with better prognosis, while high expression of the rest genes was significantly correlated with worse prognosis in AML (**Figure S1**), which confirmed the results of the univariate cox analysis.

### Development and validation of CRG signature

To avoid potential overfitting, 10 key OS-related CRGs were further screened from the 15 CRGs by minimum lambda using the LASSO regression algorithm (**Figures S2A, B**). Followed by multivariate Cox proportional hazards regression analysis, the regression coefficients of these 10 CRGs were obtained and used to develop an optimal CRG signature for the OS of patients (**Figure S2C**). Based on expression levels and regression coefficients, a patient’s risk score was defined as follows: Risk score = [Expression level of PFKL * (0.3589)] + [Expression level of IDH3G * (-0.6482)] + [Expression level of G6PD * (0.5188)] + [Expression level of DCXR * (-0.4617)] + [Expression level of CYB5R3 * (0.2328)] + [Expression level of CYB5R4 * (-0.4168)] + [Expression level of ACADS * (0.1640)] + [Expression level of MLYCD * (-0.5986)] + [Expression level of PIK3CA * (-0.2539)] + [Expression level of CDIPT * (-0.0740)].

The patients were classified into low-risk and high-risk groups according to the median risk score. The number of deaths increased with increasing risk scores, and there were more patients dead in the high -risk group than in the low-risk group (**Figures 2B, C**). With regard to expressions of the 10 model CRGs, IDH3G, G6PD, DCXR, CYB5R3, ACADS, PFKL, and CDIPT were highly expressed in the high-risk groups (**Figures 2D**, **S3A-E, G, J**), while CYB5R4, MLYCD, and PIK3CA were highly expression in the low-risk group (**Figures 5C**, **S3F, H, I**).

**Figure 3:**
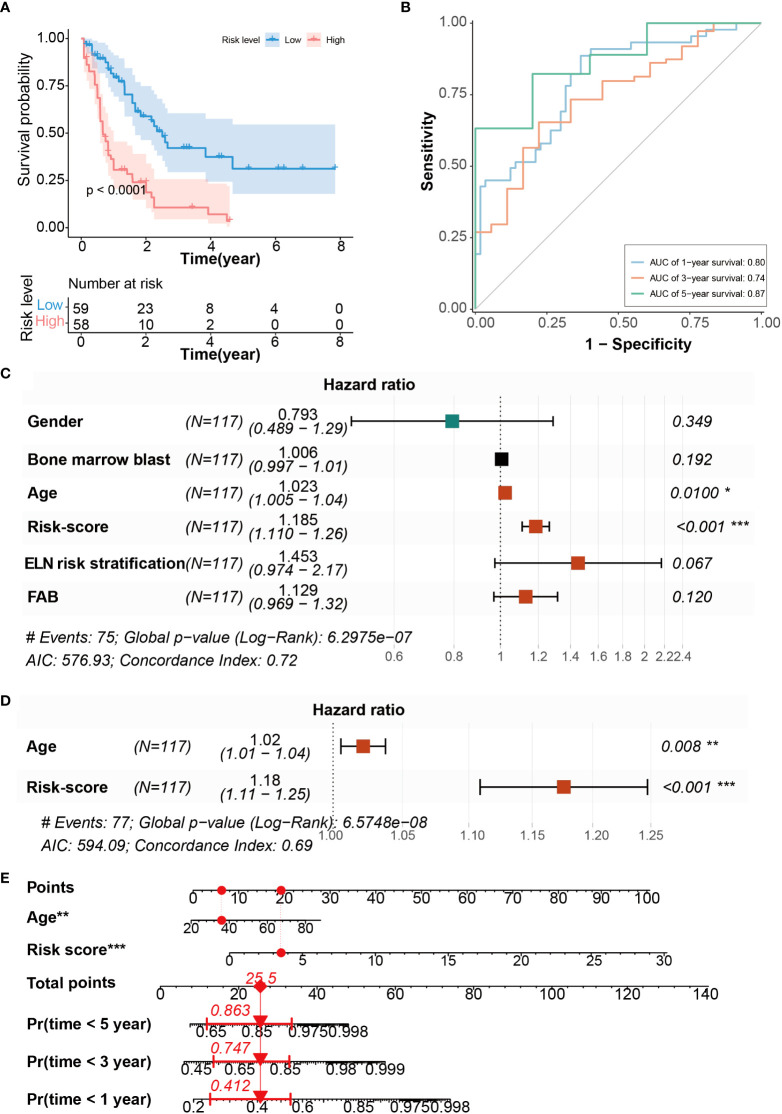
Evaluation of CRG signature to predict the OS of AML patients. **(A)** Kaplan-Meier curves of OS survival in patients with risk groups. **(B)** The AUC curves of the CRG signature for 1, 3, and 5 years. **(C)** Univariate Cox regression analysis of the risk scores and clinical parameters. **(D)** Multivariate Cox regression analysis of the risk scores and age. **(E)** Development of CRG signature clinicopathologic nomogram for predicting OS for AML patients by incorporating risk score and age. *P < 0.05, **P < 0.01, ***P < 0.001.

Next, we performed Kaplan-Meier analysis to verify the validity of the CRG signature for prognosis prediction in AML. The results showed that patients in the high-risk group had significantly shorter OS compared with those in the low-risk group (**Figure 3A**), suggesting that the CRG signature could effectively predict the prognosis of AML. Furthermore, the area under curve (AUC) of the CRG signature for 1-, 3-, and 5-year OS was 0.80, 0.74, and 0.87, respectively (**Figure 3B**). Compared with immune checkpoint-related gene signature, autophagy-related signature, and other signatures (39–42), the AUC values in CRG signature are larger at 1 year, 3 years and 5 years (**Figures S4A-C**), indicating that the CRG signature had an accurate predictive capacity for prognosis prediction in AML. We performed univariate Cox regression analysis to calculate the predictive independence of the CRG signature for AML patients, and found that age and risk score were significantly correlated with OS of the patients (**Figure 3C**). The risk score was confirmed to be an independent predictor for AML patients by the multivariate Cox regression analysis after adjusting for these clinical parameters (**Figure 3D**).

To evaluate the CRG signature more accurately, a nomogram combining the risk score and age was constructed (**Figure 3E**). The calibration curves suggested that the utility of 1- and 3-year OS could be more accurately predicted in AML patients compared with the utility of 5-year OS (**Figures S5A-C**), indicating that the integration of our risk score and age may improve OS prediction.

### External verification of the CRG signature

**Figure 4:**
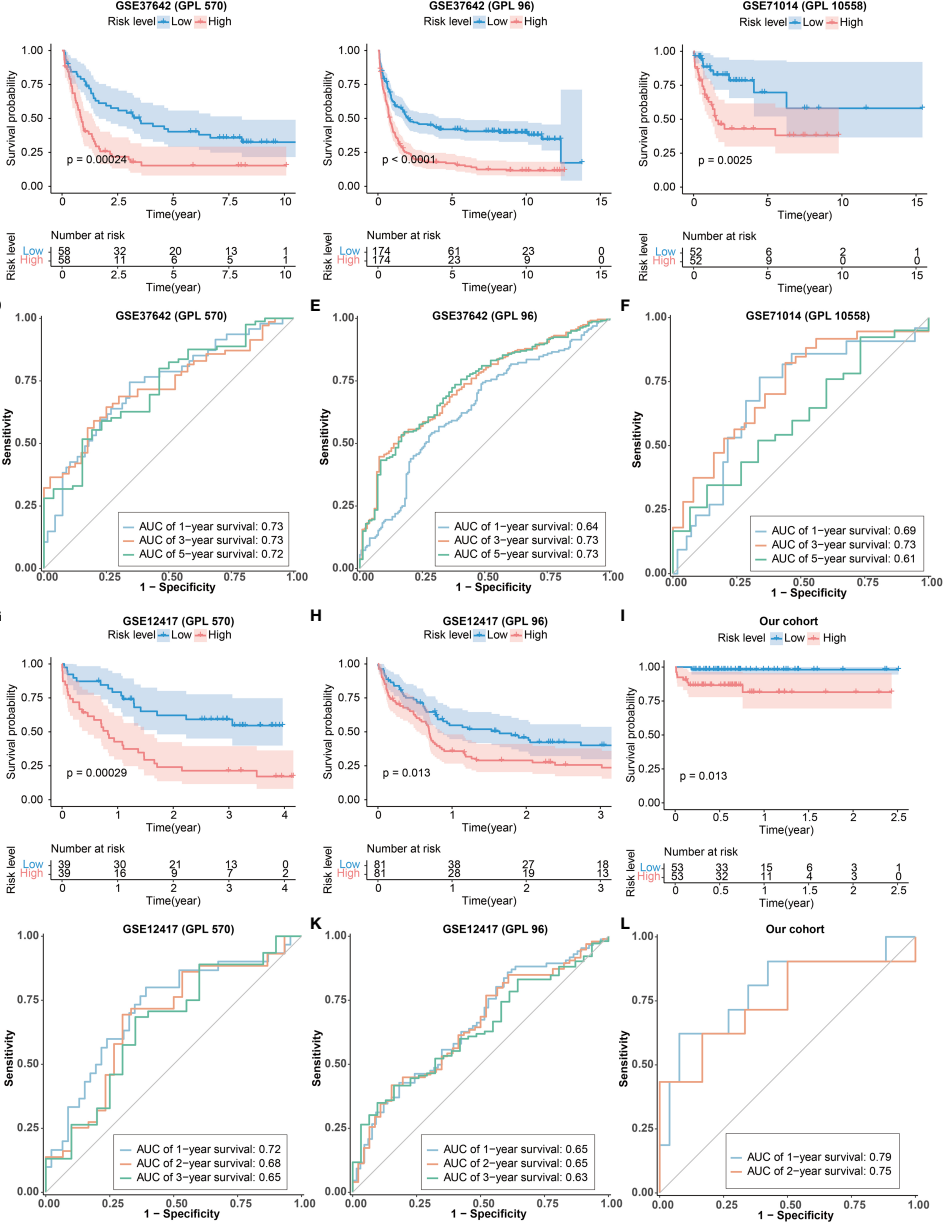
Validation of the CRG signature. **(A-C, G-I)** Kaplan–Meier curve of the CRG signature in the GEO cohorts and our cohort. **(D-F)** The ROCcurves of the CRG signature for 1-, 3-, and 5-years in the GEO cohorts. **(J, K)** The ROCcurve of the CRG signature for 1-, 2-, and 3-year in GEO cohorts. **(L)** The ROC curve of the CRG signature for 1- and 2-year in our cohort.

The scRNA-seq dataset (pbmc3k) was used to verify the expression of CRGs in specific cell types. Most of the CRGs were highly expressed in myeloid cells and hardly expressed in lymphocytes (**Figures S6A, B**), indicating that these CRGs may play a significant role in myeloid cells. To further validate the predictive utility of the CRG signature for patient OS, we calculated the risk scores for each patient in the GEO cohorts (GPL570 and GPL96 platforms of GSE37642 and GSE12417, GPL10558 platforms of GSE71014) and our own our cohort by the CRG signature formula. Patients were then divided into high- and low-risk groups according to the median risk score. Similar findings were obtained in the 6 external test sets: there were more patients alive in the low-risk group than in the high-risk group (**Figures S7-12**), and patients in the high-risk group had significantly shorter OS consistency compared with the low-risk group (**Figures 4A-C, G-I**). These results demonstrated that the CRG signature was valid in the prediction of AML prognosis. More importantly, the AUC values of the 1-, 3-, and 5-year OS in GPL570 platform of GSE37642 were 0.73, 0.73, and 0.72, respectively (**Figure 4D**). In the GPL96 platform of GSE37642, the AUC values of the 1-, 3-, and 5-year OS were 0.64, 0.73 and 0.73 (**Figure 4E**). In GSE71014, the AUC values of the 1-, 3-, and 5-year OS were 0.69, 0.73 and 0.61 (**Figure 4F**). the AUC values of the 1-, 3-, and 5-year OS in GPL570 platform of GSE12417 were 0.72, 0.68, and 0.65, respectively (**Figure 4J**). In the GPL96 platform of GSE12417, the AUC values of the 1-, 3-, and 5-year OS were 0.65, 0.65 and 0.63 (**Figure 4K**). Similar trend was observed from patients in our own cohort. The AUC values of the 1-, and 2-year OS were 0.79 and 0.75 (**Figure 4L**). The AUC values in the 6 test sets validated the accuracy of the CRG signature for predicting AML prognosis. Furthermore, the CRG signature was shown to be an independent predictor of AML patients by univariate and multivariate Cox regression analysis (**Figures S13-18**). Additionally, expression of the 10 key genes were verified in the 3 test sets, and similar expression trends were obtained (**Figures S13-15**). Overall, these results suggested that the CRG signature could be used to independently predict OS for AML patients.

### Identification and enrichment of differentially expressed genes

**Figure 5:**
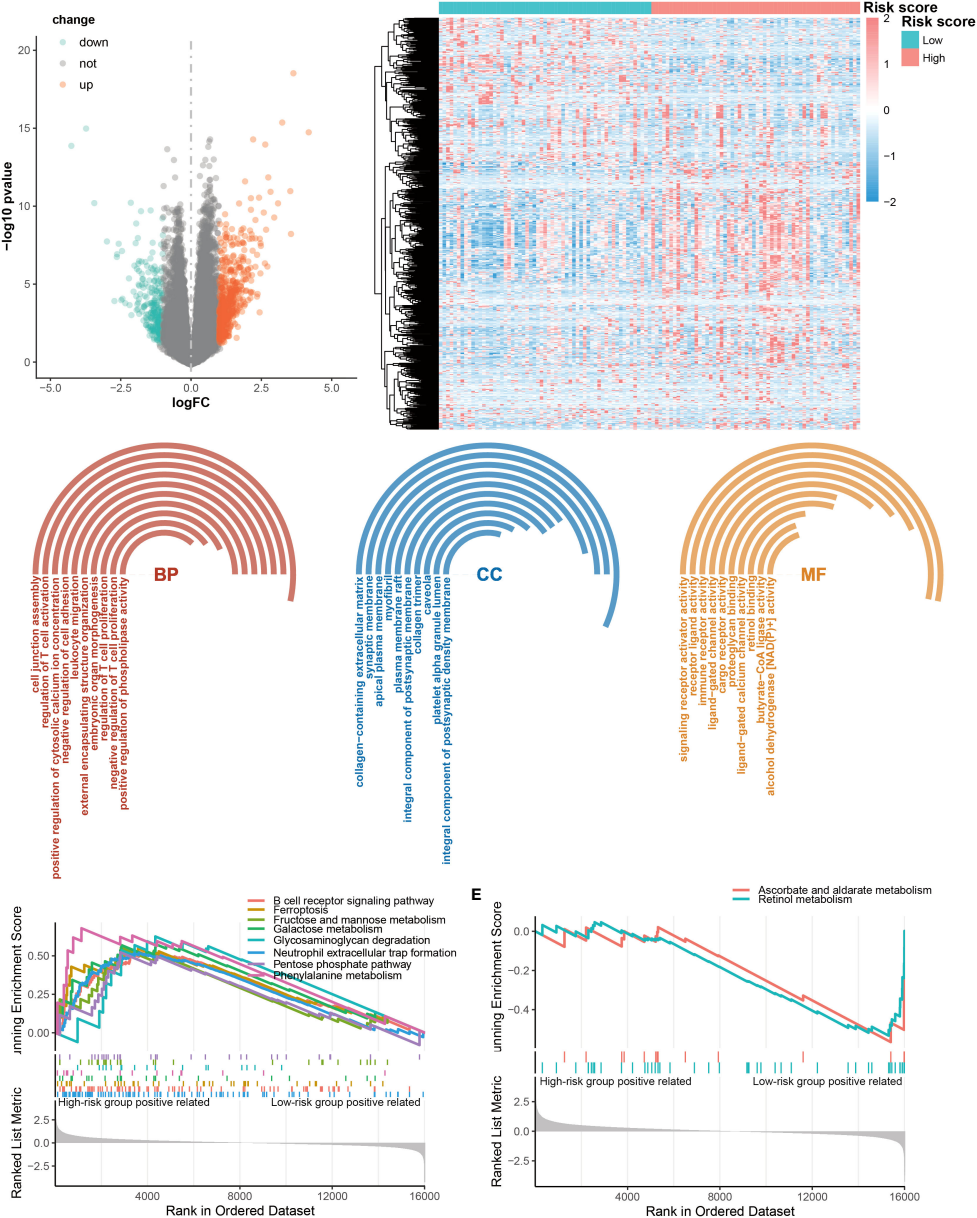
Differentially expressed genes (DEGs) between high-risk and low-risk groups. **(A)** Volcano map of DEG. **(B)** Heatmap of the DEGs. **(C)** Significantly enriched GO terms for DEG. **(D, E)** Significantly enriched DEG pathways.

To delineate the potential molecular mechanisms through which CRGs are involved in the regulation of OS in AML patients, we analyzed different gene expression patterns in the high- and low-risk groups using the DEseq2 package. We identified 699 DEGs, with 457 up-regulated genes and 242 down-regulated genes in the high-risk group compared with low-risk group (**Figures 5A, B**). The enrichment analyses of GO and GSEA showed that these DEGs were significantly enriched in immune responses and carbohydrate metabolism (**Figures 5C-E**). Moreover, the results showed that carbohydrate metabolism pathways were up-regulated in the high-risk group, and the immune response pathways were up-regulated as well (**Figure 5D**). These results suggest that CRG signature may play significant roles in the activation of immune responses in AML.

### Immune cell proportion and correlation analyses for different risk groups

**Figure 6:**
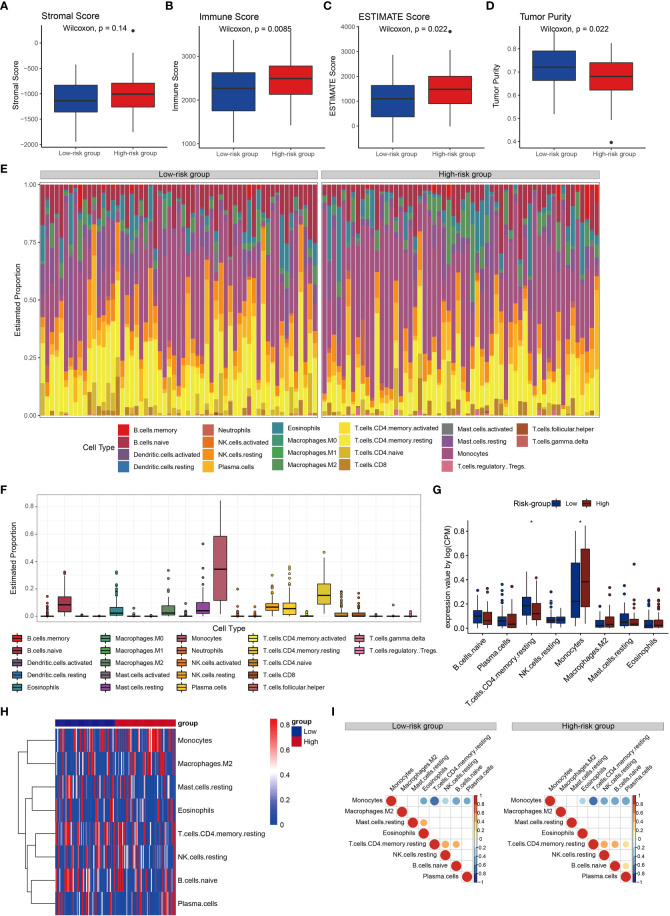
Immune cell proportion and correlations analyses for the risk groups. **(A-D)** Stromal score, immune score, estimate score and tumor purity of the risk groups. **(E)** Relative proportions of immune cell in the high- and low-risk groups. **(F)** Boxplots illustrate the 22 immune cell proportions in the TCGA cohort. **(G)** Boxplots illustrate the 22 immune cell proportions between high- and low-risk groups. Statistical significance at the level of ∗< 0.05. **(H)** Heatmap of the immune cells for patients with high and low high- and low-risk groups. Only the immune cells in which the nonzero proportions in all samples exceeded half were retained. **(I)** Correlations between immune cells in risk groups.

To further explore the immune status of high- and low-risk groups, we calculated stromal score, immune score and estimate score by ESTIMAT software, and found that they were higher in the high-risk group than those of low-risk group, suggesting that there were more immune infiltrating cells and stromal cells in the microenvironment of the high-risk group than the low-risk group. (**Figures 6A-C**). Compared to the low-risk group, the tumor purity was lower in high-risk group (**Figure 6D**), suggesting that AML with low tumor purity had a worse prognosis. These results implied that the CRG signature was closely related to the immune microenvironment.

Subsequently, we investigated immune cell properties of 22 immune cell types in the training set (**Figures 6E–F**) her by using the CIBERSORT algorithm of 1000 permutations (43). We found that the proportion of resting memory CD4+ T cells in the low-risk group was significantly higher than that in the high-risk group, while the proportion of monocytes in the low-risk group was significantly lower than that in the high-risk group (**Figures 6G, H**). Monocytes were negatively associated with eosinophils, resting memory CD4+ T cells, resting NK cells, naïve B cells, and plasma cells, and resting memory CD4+ T cells was positively correlated with resting NK cells, and naïve B cells in both groups (**Figure 10I**). Resting memory CD4+ T cells were positively correlated with eosinophils in the low-risk group (**Figure 6I**). Resting mast cells were positively correlated with eosinophils in the low-risk group, while plasma cells were positively correlated with resting memory CD4+ T cells and naïve B cells in the high-risk group (**Figure 6I**). These results confirmed that in AML the CRG signature played significant roles in immune responses.

### Mutation characterization for risk groups

**Figure 7:**
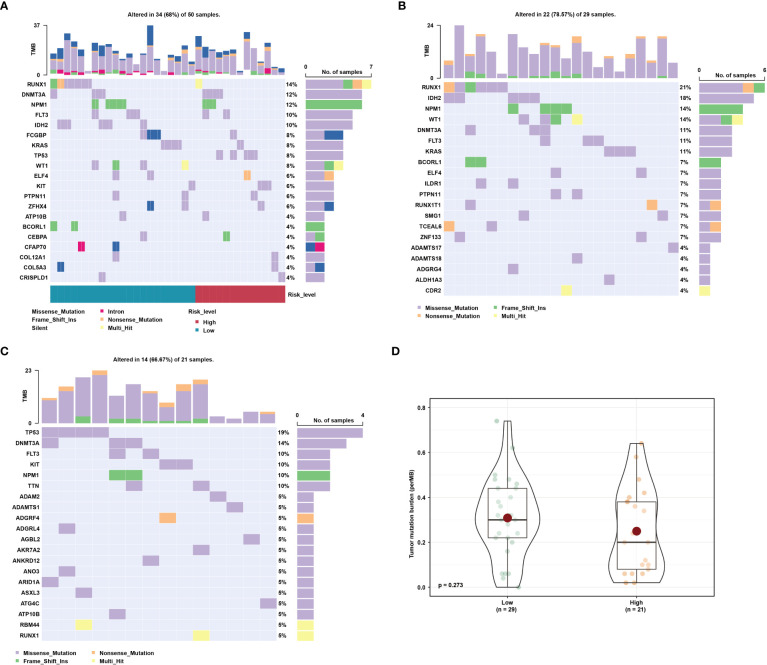
Mutation characterization for different risk groups. **(A)** Gene mutations in AML patients. **(B)** Gene mutations in low-risk group. **(C)** Gene mutations in high-risk group. **(D)** Tumor mutational burden between high- and low-risk groups.

To investigate relationship between the CRG signature and AML mutations, we analyzed SNP profiles using the Maftools R package. The most common type of gene mutation in high- and low-risk groups was missense mutation (**Figures 7A-C**). Gene mutations of patients in the high-risk and low-risk groups were different. The incidence of NPM1, DNMT3A and FLT3 mutations was high in both groups (**Figures 7A-C**). However, RUNX1, IDH2, WT1, and KRAS mutations were more frequently in the low-risk group (**Figure 7B**), and TP53, KIT, and TTN mutations were more common in the high-risk group (**Figure 7C**). Compared with the high-risk group, patients in the low-risk group had a higher tumor mutational burden (**Figure 7D**).

### Potential drug susceptibility

**Figure 8:**
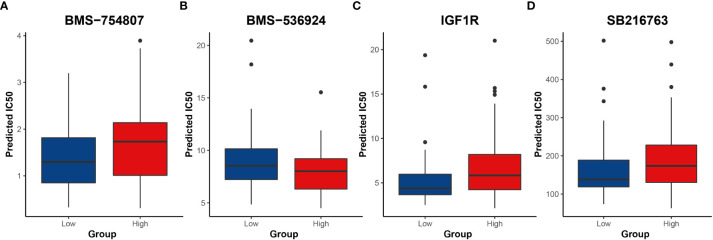
Results of potential drugs for risk groups **(A-D)** Inhibitors of carbohydrate metabolism. IC50 indicates the half maximal inhibitory concentration.

Next, potential clinical responses to chemotherapy based on the CRG signature was calculated by the oncoPredict R package. The predicting drug response function of oncoPredict R package implements the pipeline for the prediction of clinical chemotherapeutic response by using only baseline tumor gene expression data (25), and drugs with a P value less than 0.05 were selected (**Figure S25**). These drugs mainly targeted the PI3K/AKT/mTOR signaling pathway, carbohydrate metabolism, DNA synthesis and damage, epigenetics and apoptosis (**Figure 8**, **Figure S26-29**). The low-risk group was more sensitive to most of the carbohydrate metabolism inhibitors (**Figures 8A-D**).

## Discussion

Targeted therapy and immunotherapy have played crucial roles in the treatment of AML patients, but the 5-year survival rate of AML remains disappointing due to high relapse rate. By classifying patients into high- or low-risk group based on robust RNA-seq and clinical data signatures, accurate prediction of prognosis and drug response may improve physicians’ decision-making ability to select personalized treatment. In this study, OS-related CRGs were identified by using profiles from TCGA AML patients, and a CRG signature consisted of 10 CRGs that can accurately evaluate the OS of AML patients was developed. The validation results of GEO database and our data confirmed that the CRG signature was a steady and independent predictor for the risk stratification of AML patients. In addition, we investigated the clinical relevance and integrated risk groups with underlying gene expression programs that can play significant roles in AML biology, distinct tumor-immune infiltrating landscapes, clinical outcome, and potential drug susceptibility.

The complicated carbohydrate metabolism-related mechanism assembled by large amounts of proteins with clear functions plays a critical role in maintaining essential functions and homeostasis of cells (10). Carbohydrate metabolism-related activities in cancer cells provide sufficient energy and metabolic substrates for cell proliferation and division. The disordered carbohydrate metabolism can be a primary event in oncogenesis, and many carbohydrate metabolism-related oncogenes and signaling pathways have been considered as key regulators in tumor progression (44–46). One well-studied link between tumorigenesis and carbohydrate metabolism is the phosphoinositide 3-kinase (PI3K)/serine and threonine kinase AKT/mammalian target of rapamycin (mTOR) signaling pathway, which upregulates glucose intake and metabolism *via* various mechanisms (47, 48). In addition, carbohydrate metabolism is positively regulated by KRAS and MYC (49), and negatively regulated by P53 (44). Drugs targeting carbohydrate metabolism- related oncogenes and pathways have been applied in cancer treatment (44). APR-246 was a safe p53 wild-type restoration compound with a favorable pharmacokinetic profile, and it was found glucuronidation metabolites increased in a phase II clinical trial after injection 1-2 hours (50). Carbohydrate metabolism has been highlighted in AML and is a valuable potential target for therapy. Many molecular targets and carbohydrate metabolism inhibitors have been identified (21, 44, 51). However, the relationship between carbohydrate metabolism and prognosis was rarely reported in AML. Our analysis of CRGs in AML contributed to the discovery of additional prognostic biomarkers and classification of high- and low-risk patients.

Transcriptome-based gene profiling is a promising approach to monitor prognostic risk in cancer (52). Signatures related to the prognosis of AML have been developed (53). However, the CRG signature in AML has rarely been developed so far. Given our discovery of important roles for CRGs in AML OS and the key role of carbohydrate metabolism in cancers, we developed a CRG signature composed of 10 OS-related CRGs to evaluate patients’ prognosis. It was found that patients in the low-risk group had significantly longer OS than those in the high-risk group. Additionally, the AUC values of the ROC curves for 1-year, 3-year and 5-year OS were 0.80, 0.74 and 0.87, respectively. As an independent predictor, the CRG signature was verified not only in public datasets, but also in AML data collected at our center. These results indicate that the CRG signature was a stable and reliable model to evaluate prognosis in AML. A nomogram integrating risk score and age further provided the possibility of individualized utility to monitor patients’ outcomes.

AML has high genetic heterogeneity, and gene mutation plays a pivotal role in the occurrence, development, treatment and prognosis of AML (54). So, we analyzed gene mutation status of high- and low-risk groups. Somatic heterozygous RUNX1 mutations occurred in approximately 10-32% of AML patients and were associated with unfavorable prognosis (55, 56). The overall RUNX1 mutation rate in AML patients in our study was 14%, which is consistent with the previous reports. RUNX1 mutation was the most common mutations in the low-risk group (21%), while the mutation rate of TP53 (19%) was higher than other mutations in high-risk group. It was reported that about 13% of AML patients are accompanied by TP53 mutation, and the prognosis with TP53 mutation was poor (57). In addition, mutations in IDH2, FLT3, and DNMT3A were present in 10%, 30%, and 20.9% of AML patients, respectively (58–60). And these mutations were common in both risk groups in our study. It was found that AML patients with NPM1 mutation belong to the (European LeukmiaNet) ELN favorable risk category (61). In this study, similar result was obtained that the incidence of NPM1 mutations was higher in low-risk group compared to the high-risk group. These results validated the reliability of the CRG signature.

We identified differentially expressed genes in high- and low-risk groups which were significantly enriched in regulation of immune responses, including T cell activation and proliferation, leukocyte migration, receptor ligand activity, immune receptor activity, ligand−gated channel activity, and cargo receptor activity. The related pathways mainly focused on B cell receptor signaling pathway, neutrophil extracellular trap (NET) formation pentose phosphate pathway, and carbohydrate metabolism (fructose and mannose metabolism, galactose metabolism, glycosaminoglycan degradation). These results revealed that carbohydrate metabolism may play a crucial role in the immune status of high- and low-risk groups. B cell receptor (BCR) signaling pathway was crucial for immunity by recognizing a variety of antigen and passing relevant signals (62). Inhibition of the key modulation factor of the B cell receptor signaling pathway have been proven to be an effective therapeutic approach for AML (63). In addition, NET has been implicated in a variety of pathologies from autoimmunity to cancer, and its release was impaired in AML (64). It may contribute to the increased susceptibility of this population (64). CAR-T cell therapy has been used for the treatment of AML (65), which induces T cell activation, proliferation, and effector function through the binding of reprogrammed T cells to relevant antigens on the surface of tumor cells, ultimately leading to tumor cell death (66). Interestingly, T cell activation and proliferation were observed in our study, which suggested that the CRG signature may be helpful to assess the prognosis immunotherapy of AML. A growing body of preclinical studies have shown that tumor-infiltrating lymphocytes (TILs) have a large impact on disease progression, response to therapy, and prognosis in many cancers (67–69). It was found that there were more immune infiltrating cells and stromal cells in the microenvironment of the high-risk group than the low-risk group by the stromal score, immune score and estimate score. In addition to CAR-T, the current main immunotherapy methods for AML include naked antibodies and conjugated monoclonal antibodies, immune checkpoint inhibitors, and bispecific T cell-engaging antibodies (70). These immunotherapy methods mainly target CD7, CD33, CD45, CD56, CD133, CD123 and CLEC12A (70–73). Immune checkpoints inhibitors mainly target CTLA4, PD1/PD-L1. Through different targets, these immunotherapies resulting in tumor cells being attacked and disrupted by cytotoxic agents or the patient’s own activated immune system. However, the proportion analysis of tumor-infiltrating immune cells revealed a significantly lower abundance of resting CD4+ T memory cells in the high-risk group. These results suggest that, based on the CRG signature, the suppressed immune microenvironment potentially contribute to poor responses to immunotherapy in high-risk patients. However, the proportion analysis of tumor-infiltrating immune cells revealed a significantly lower abundance of resting CD4^+^ T memory cells in the high-risk group. These results suggest that, based on the CRG signature, the suppressed immune microenvironment potentially contribute to poor responses to immunotherapy in high-risk patients.

The ‘oncoPredict’ R package was used to assess clinical drug response in AML patients. High- and low-risk groups responded differently to chemotherapy drugs. These drugs mainly focused on PI3K/AKT/mTOR signaling pathway and carbohydrate metabolism. About one-fifth of these drugs were inhibitors of PI3K/AKT/mTOR signaling pathway. These inhibitors (74–76) were effective for AML of both high- and low-risk groups, and most of them had lower IC_50_ in high-risk groups, suggesting that inhibition of the PI3K signaling pathway may provide a new potential approach for AML treatment. At present, pre-clinical studies have shown that these inhibitors could reduce the proliferation and induce apoptosis, and reverse the multidrug resistance of AML (77–79). Therapeutic effect of these inhibitors in AML confirmed our previous inference that the PI3K/AKT/mTOR signaling pathway was activated in AML. In addition, AML patients were sensitive to carbohydrate metabolism inhibitors. Previous research found that inhibition of insulin receptor isoform A and insulin-like growth factor-1 receptor can inhibit proliferation and promote apoptosis in AML (80). It was reported GSK3 inhibition primes a pro-differentiative/apoptotic transcription program (81). Our results and these reports implied that inhibition of carbohydrate metabolism may become a new breakthrough in the treatment of AML. However, more in-depth work is needed to explore and verify this observation.

Our study systematically analyzed carbohydrate metabolism-related transcriptomic profiles and developed a risk-prognostic signature based on survival-related CRGs in AML patients. We validated the CRG signature in publicly accessible, retrospective datasets, and used our own independent external validation to assess its potential clinical relevance. Based on the CRG signature, mutations, immune, and drug sensitivity were analyzed. The results showed that the CRG signature was reliable and may provide theoretical support for AML prognostic judgment and treatment. Our research mainly focused on clinical bulk gene expression data. Further work is required to fully understand the role of CRG in AML. Combined scRNA-seq data with clinical bulk gene expression data, a study had developed a computational pipeline for identifying the prognostic and predictive signature that connects cancer cells and microenvironmental cells (82, 83). It can help us gain a more comprehensive understanding of the role of the CRG signature.

## Data availability statement

The original contributions presented in the study are included in the article/**Supplementary Material**. Further inquiries can be directed to the corresponding authors.

## Ethics statement

The studies involving human participants were reviewed and approved by the Affiliated Hospital of Southwest Medical University. Written informed consent for participation was not required for this study in accordance with the national legislation and the institutional requirements.

## Author contributions

YoY, LG, HL, and WL contributed to the conception and design of the experiment. YoY, YaY, JL, JG and YZ performed the study and participated in data acquisition. YoY, YaY, QG and YZ contributed to the clinical bulk gene expression data analysis and interpretation. YoY, YaY, LG, HL and WL wrote and revised the paper. LG, HL and WL were the guarantors of this work. All authors approved the final version of the paper. YoY, YaY and JL contributed equally to this paper.

## Funding

This work was supported by grants from Medical Science and Technology Project of Health Commission of Sichuan Province (No.21PJ091); the Special Project of Science and Technology Research of Sichuan Administration of Traditional Chinese Medicine (2020JC0135); the Applied Basic Research Project of Southwest Medical University (2021ZKQN083); the Major Science and Technology Projects in Sichuan Province (2019YFS0531); Science and Technology Strategic Cooperation Project of Luzhou Municipal People’s Government-Southwest Medical University-Applied Basic Research Project (2021LZXNYD-J22); Sichuan Science and Technology Program (22ZDYF3802); Doctoral Research Initiation Fund of Affiliated Hospital of Southwest Medical University; Natural Science Foundation of Sichuan Province (2022NSFSC0723); Natural Science Foundation of Shandong Province (ZR2021YQ50); Southwest Medical University Program 2022ZD007.

## Acknowlegments

We thanked Dr.Jianming Zeng (University of Macau), and all the members of his bioinformatics team, biotrainee, for generously sharing their experience and assisting us with the analysis of scRNA-seq data.

## Conflict of interest

The authors declare that the research was conducted in the absence of any commercial or financial relationships that could be construed as a potential conflict of interest.

## Publisher’s note

All claims expressed in this article are solely those of the authors and do not necessarily represent those of their affiliated organizations, or those of the publisher, the editors and the reviewers. Any product that may be evaluated in this article, or claim that may be made by its manufacturer, is not guaranteed or endorsed by the publisher.
