# Supplementary material for: Establishment and validation of a carbohydrate metabolism-related gene signature for prognostic model and immune response in acute myeloid leukemia
Source: Front Immunol. 2022 Dec 5;13:1038570. doi: 10.3389/fimmu.2022.1038570 (PMC9761472; doi:10.3389/fimmu.2022.1038570)
Supplement: Supplementary file 1 [file Image_1.pdf]

Supplementary figures for a carbohydrate metabolism-related gene signature for the prediction of prognosis and therapeutic response in acute myeloid leukemia

You Yang[1,†], Yan Yang[1,†], Jing Liu[1,†], Yan Zeng[1], Qulian Guo[1], Jing Guo[2], Ling Guo[1,*], Haiquan Lu[3,*], Wenjun Liu[1,*]

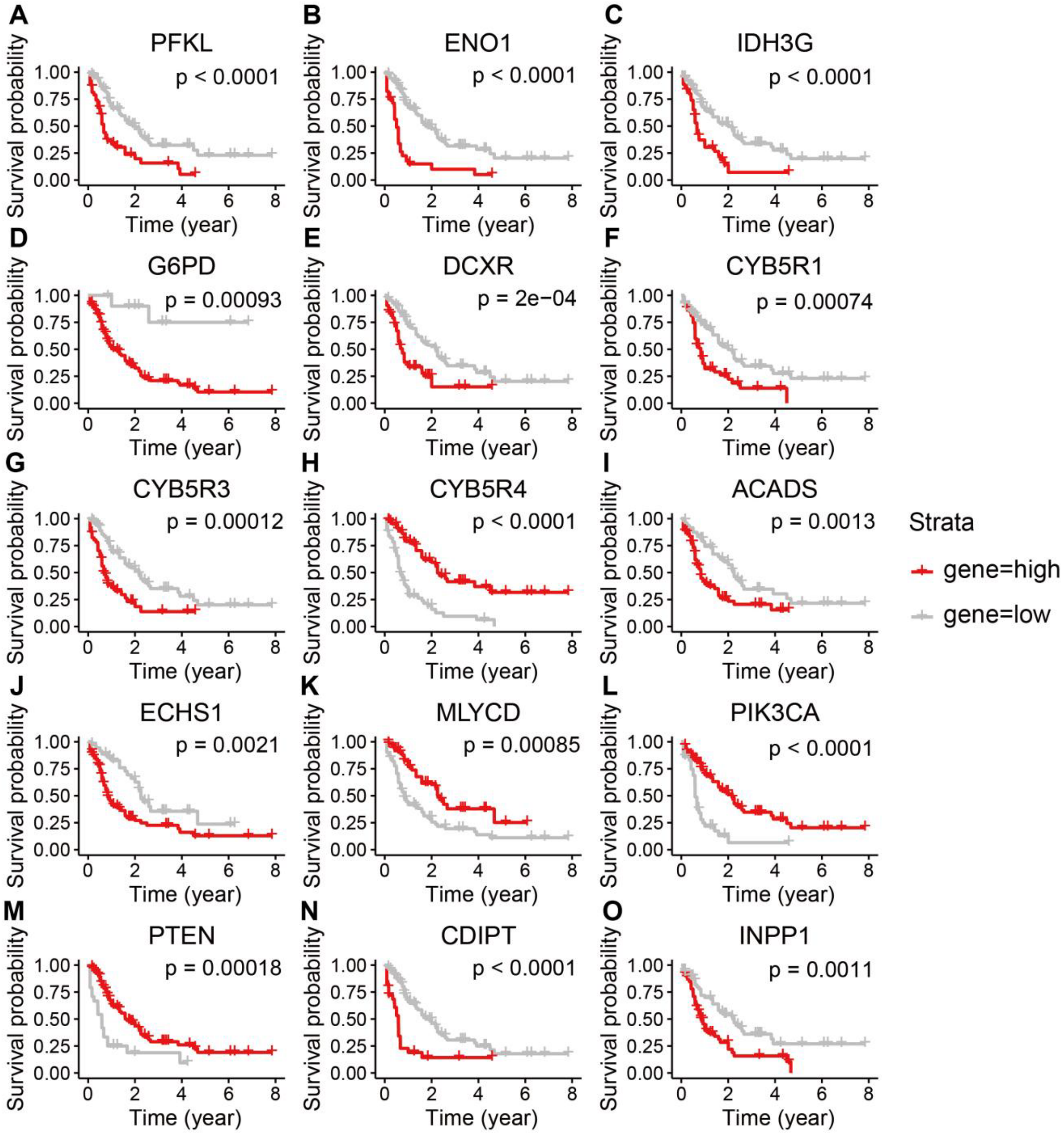

Supplementary Figure S1. Kaplan-Meier curves of OS for 15 CRGs

Kaplan-Meier analyses of overall survival were performed based on clinical and molecular data from 117 AML patients. The patients were stratified by indicated mRNA levels in the primary tumor, which were greater (red) or less (grey) than the median level. P value (log-rank test) are shown.

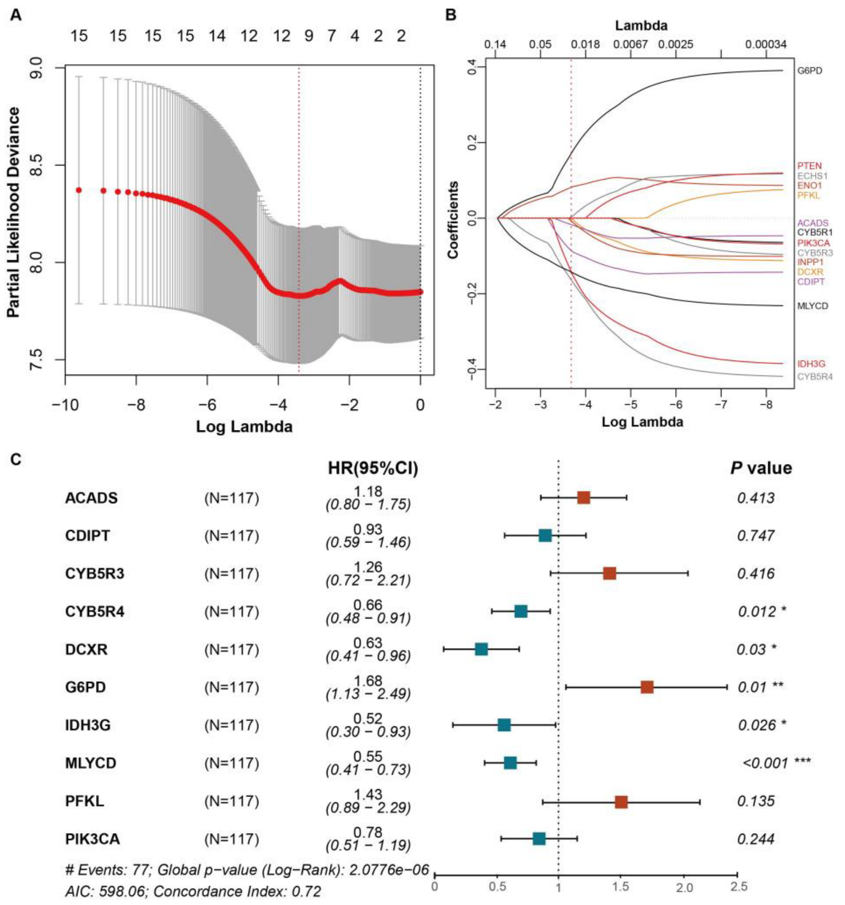

Supplementary Figure S2. Identification and development of a CRG signature for overall survival in AML cohort.

(A) Cross-validation for tuning parameter selection in the proportional hazards model. (B) LASSO coefficient spectrum of 10 CRGs in AML. (C) The hazard ratio of 10 model CRGs. HR indicates hazard ratio; CI indicates confidence interval.

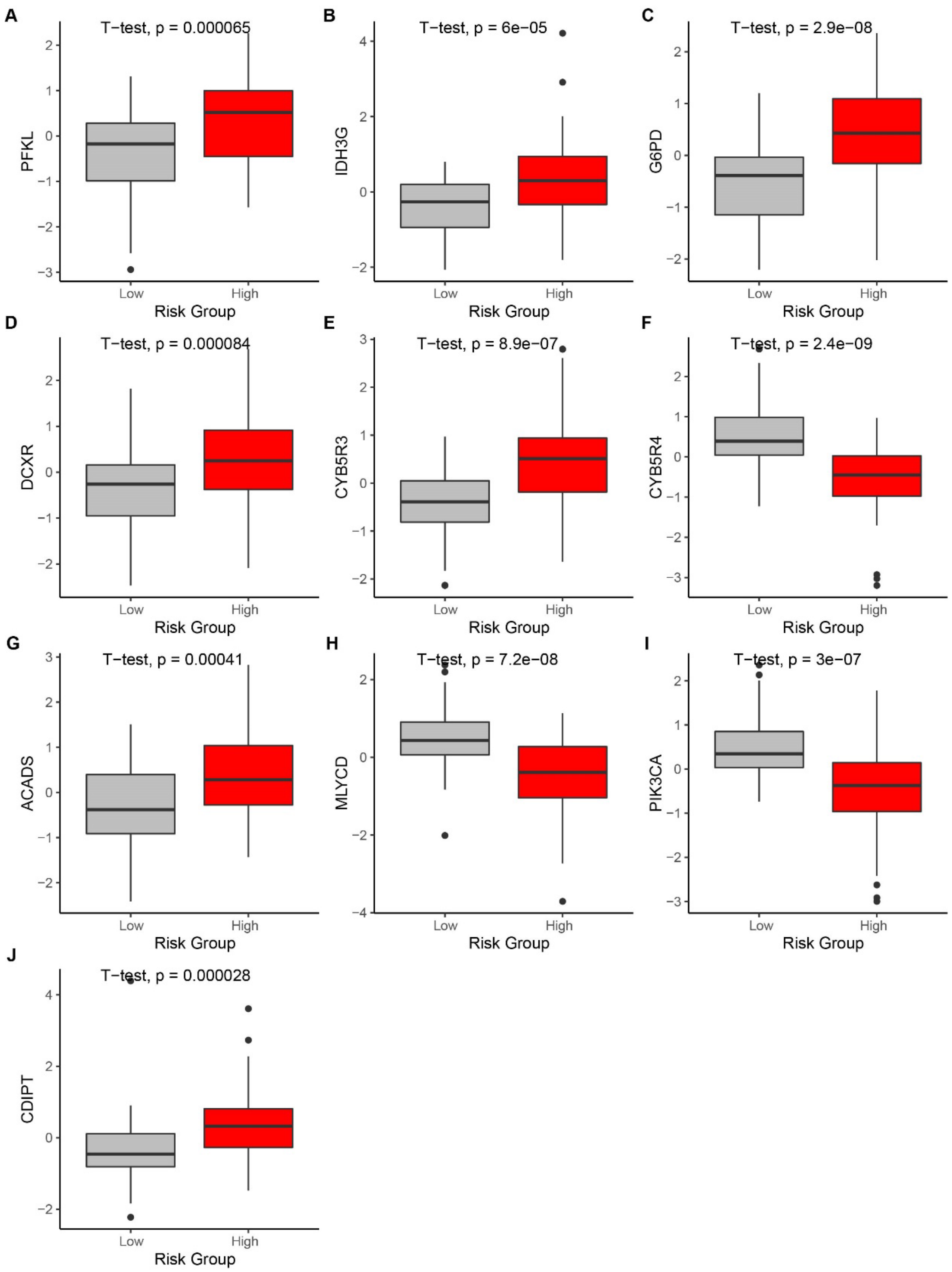

Supplementary Figure S3. Relative mRNA expression levels of 10 CRGs in high and low risk groups (training set)

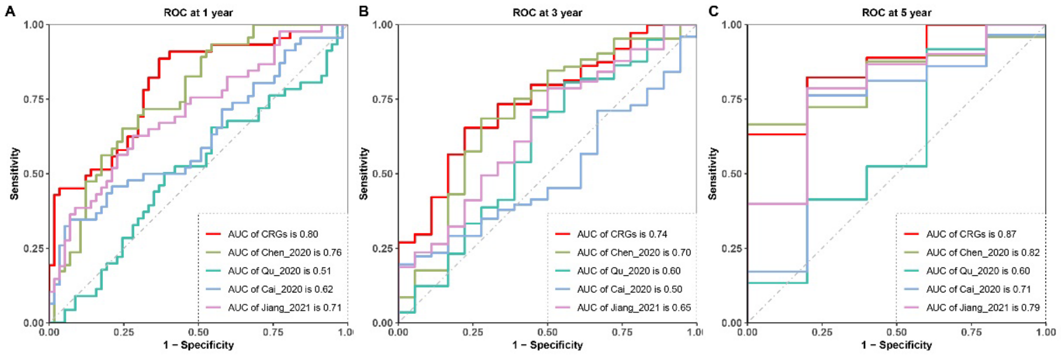

Supplementary Figure S4. The ROC curves of signatures in AML

(A) The ROC curves of signatures in AML for 1-year; (B)The ROC curves of signatures in AML for 3-year; (C) The ROC curves of signatures in AML for 5-year

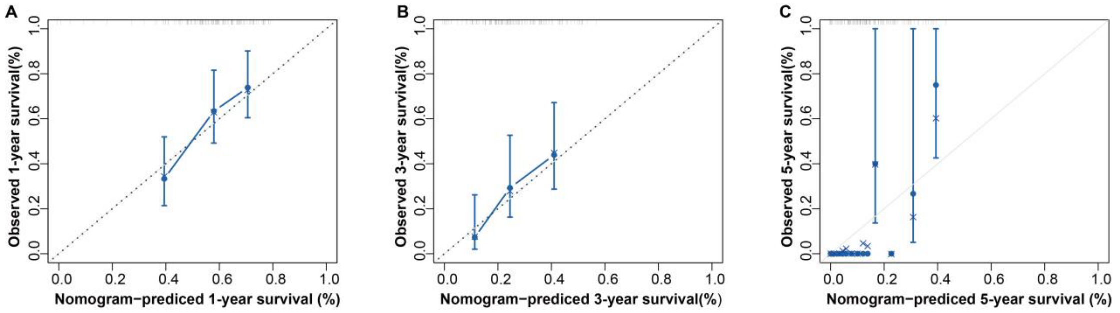

Supplementary Figure S5 Calibration curves of the autophagy clinicopathologic nomogram-predicted and observed 1-, 3- and 5-year survival of AML patients.

The dashed line represents the ideal performance, and the actual performance of the CRG signature is represented by the blue lines.

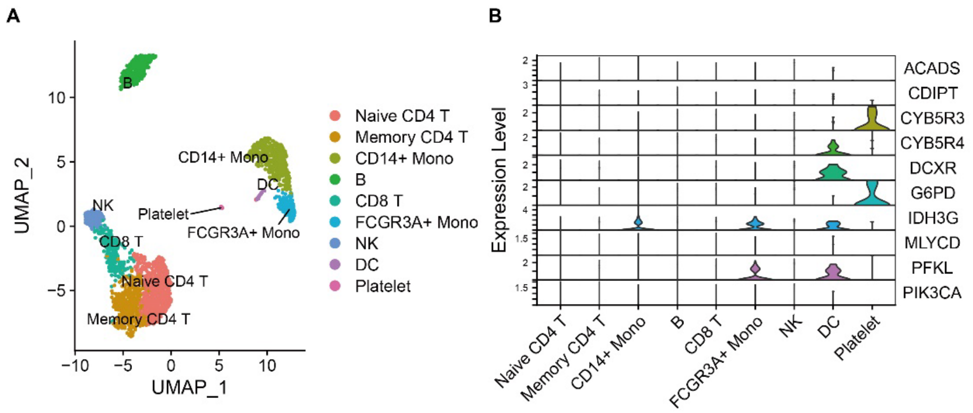

Supplementary Figure S6 Expression of CRGs in specific cell types

(A) Cell types in peripheral blood; (B) Expression of CRGs in specific cell types.

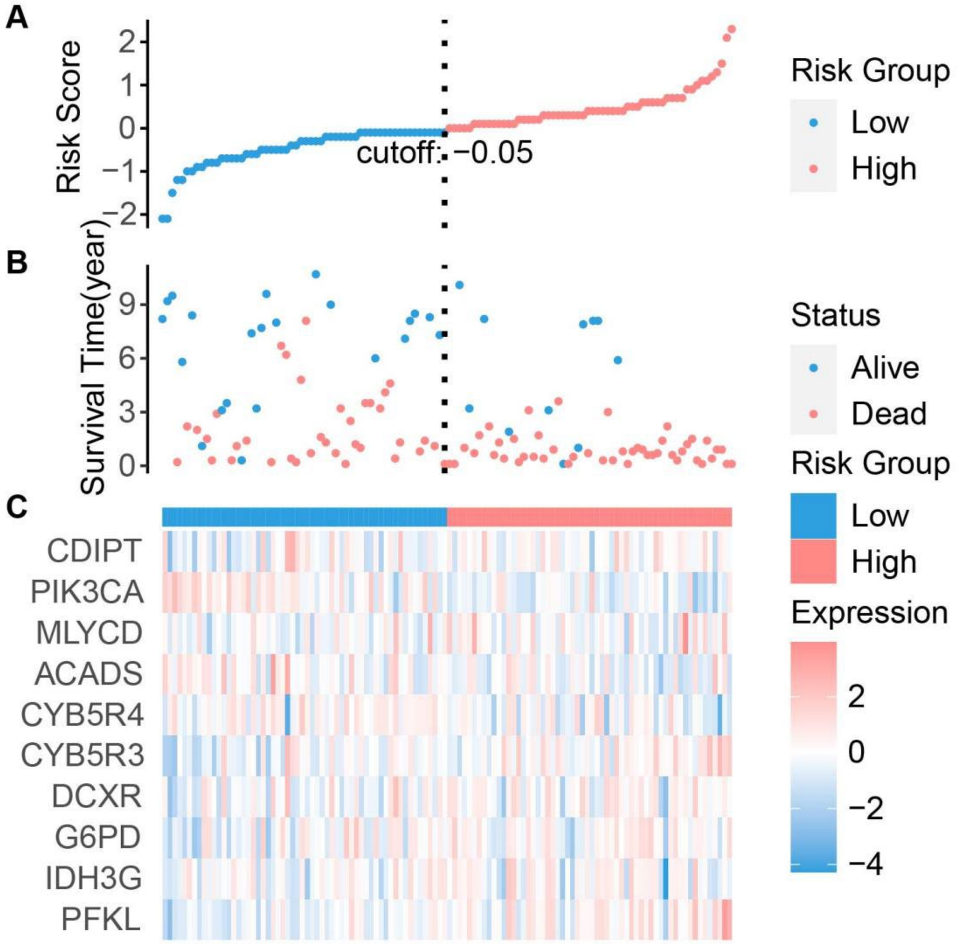

Supplementary Figure S7. Prognostic analysis of the CRA-related gene signature in the test set (GSE37642, GPL570).

(A) Risk Groups based on patients' risk scores. (B) Patients' survival status along with their risk scores. (C) The expressions of the 10 model CRGs in the high- and low-risk groups.

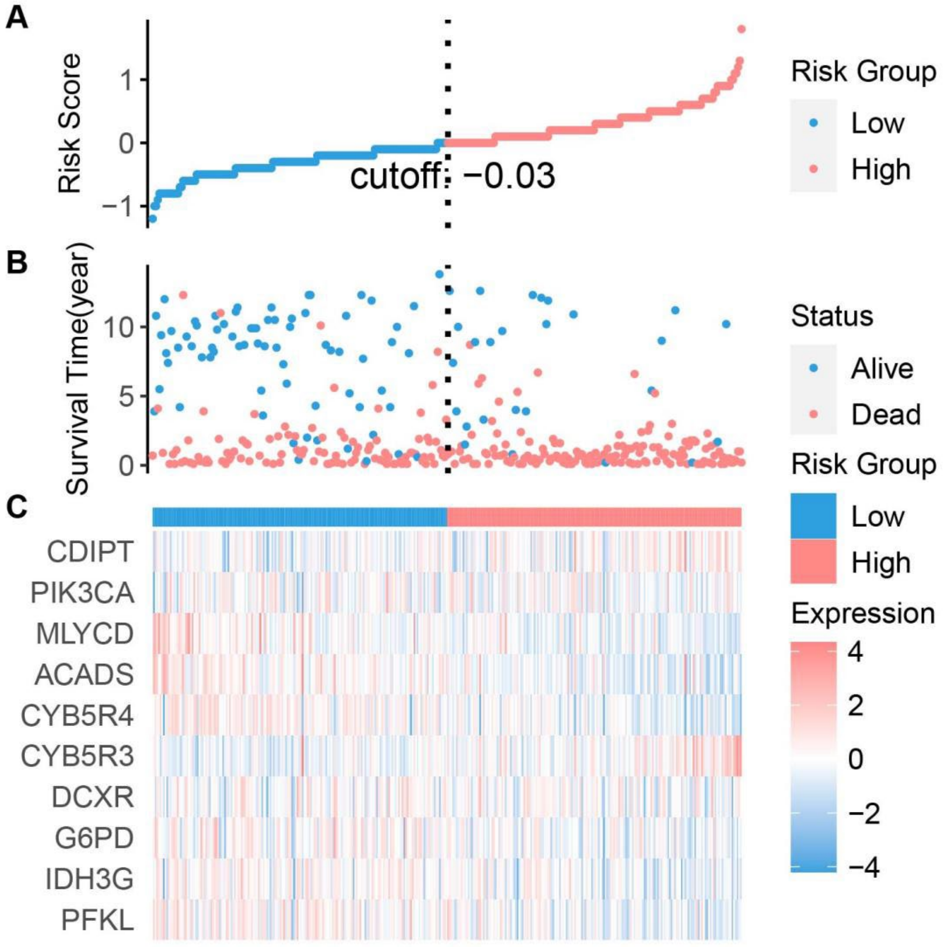

Supplementary Figure S8. Prognostic analysis of the CRA-related gene signature in the test set (GSE37642, GPL96).

(A) Risk Groups based on patients' risk scores. (B) Patients' survival status along with their risk scores. (C) The expressions of the 10 model CRGs in the high- and low-risk groups.

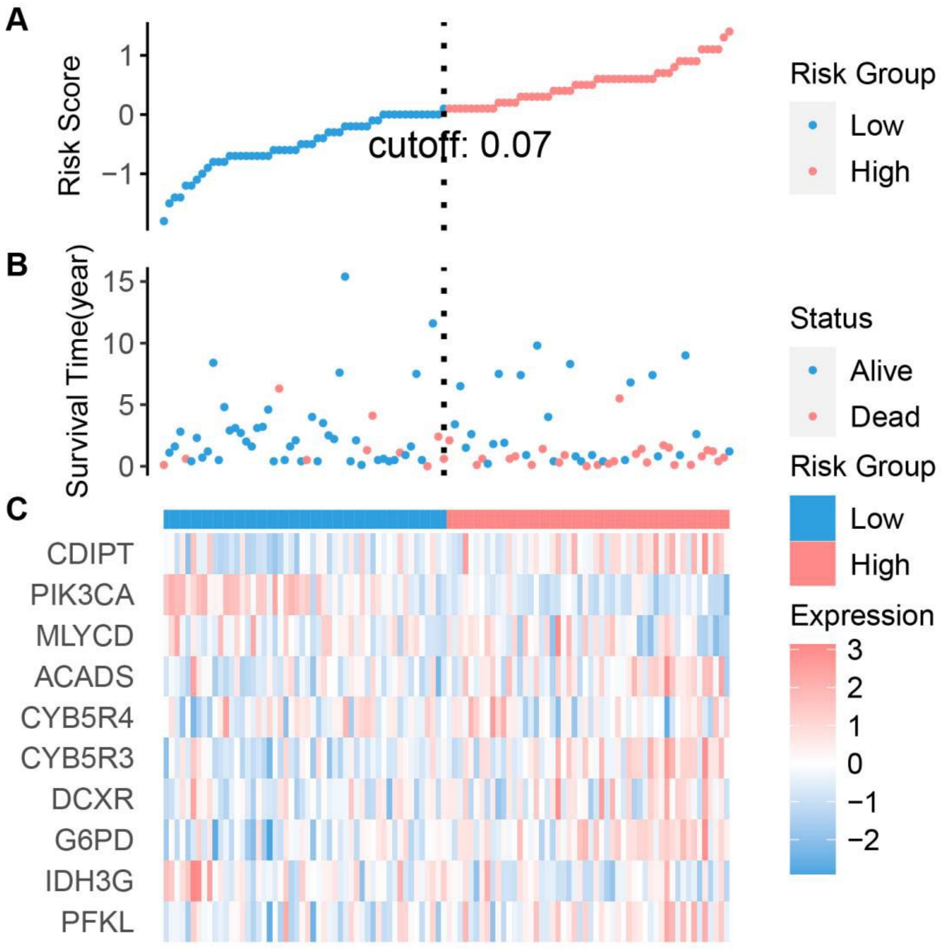

Supplementary Figure S9. Prognostic analysis of the CRA-related gene signature in the test set (GSE71014, GPL10558).

(A) Risk Groups based on patients' risk scores. (B) Patients' survival status along with their risk scores. (C) The expressions of the 10 model CRGs in the high- and low-risk groups.

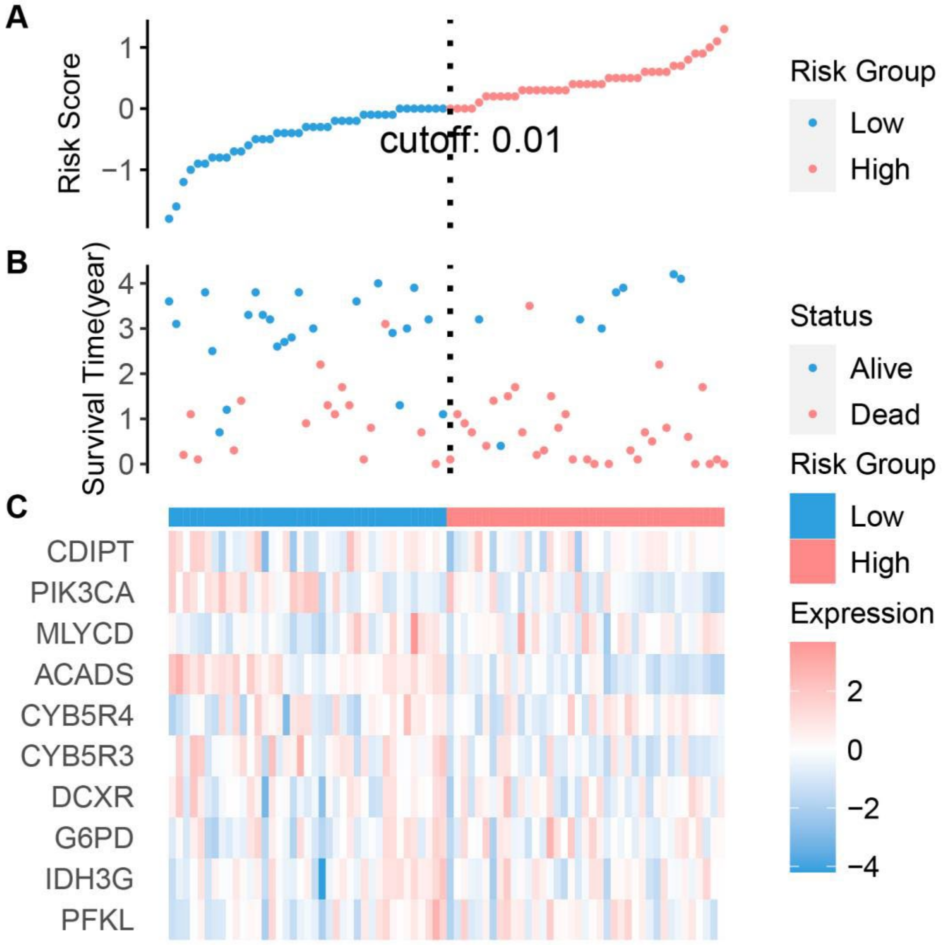

Supplementary Figure S10. Prognostic analysis of the CRA-related gene signature in the test set (GSE12417, GPL570).

(A) Risk Groups based on patients' risk scores. (B) Patients' survival status along with their risk scores. (C) The expressions of the 10 model CRGs in the high- and low-risk groups.

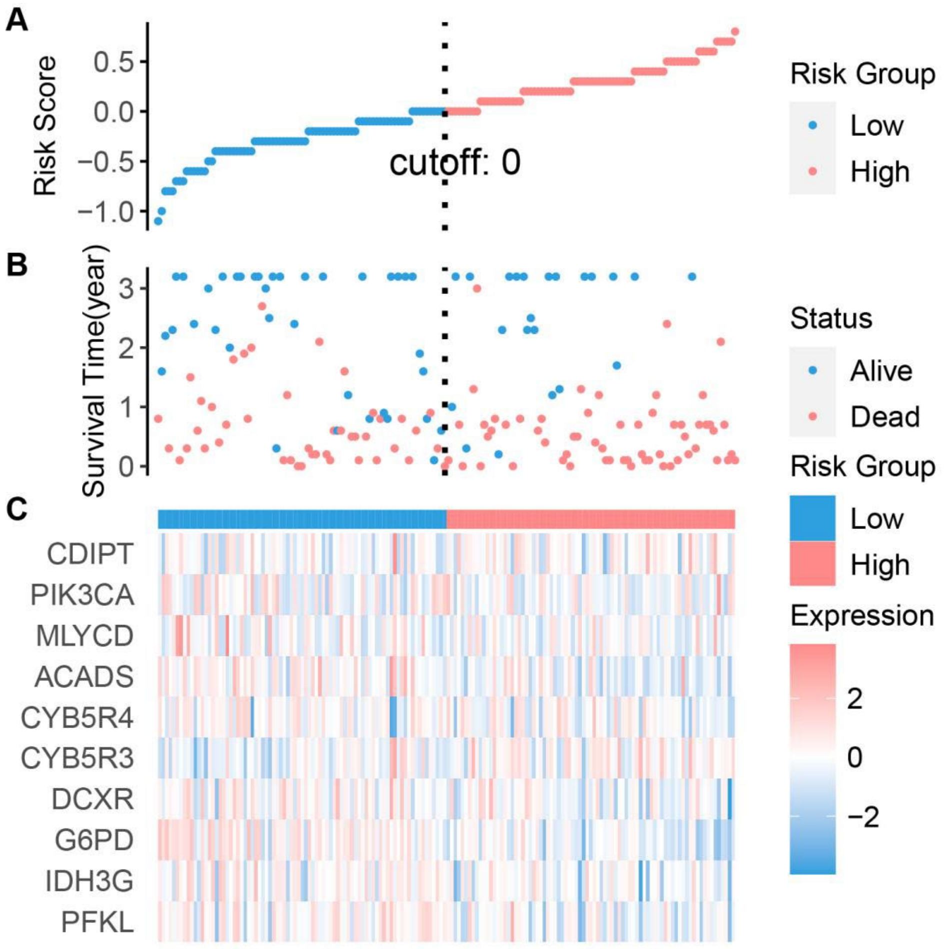

Supplementary Figure S11. Prognostic analysis of the CRA-related gene signature in the test set (GSE12417, GPL96).

(A) Risk Groups based on patients' risk scores. (B) Patients' survival status along with their risk scores. (C) The expressions of the 10 model CRGs in the high- and low-risk groups.

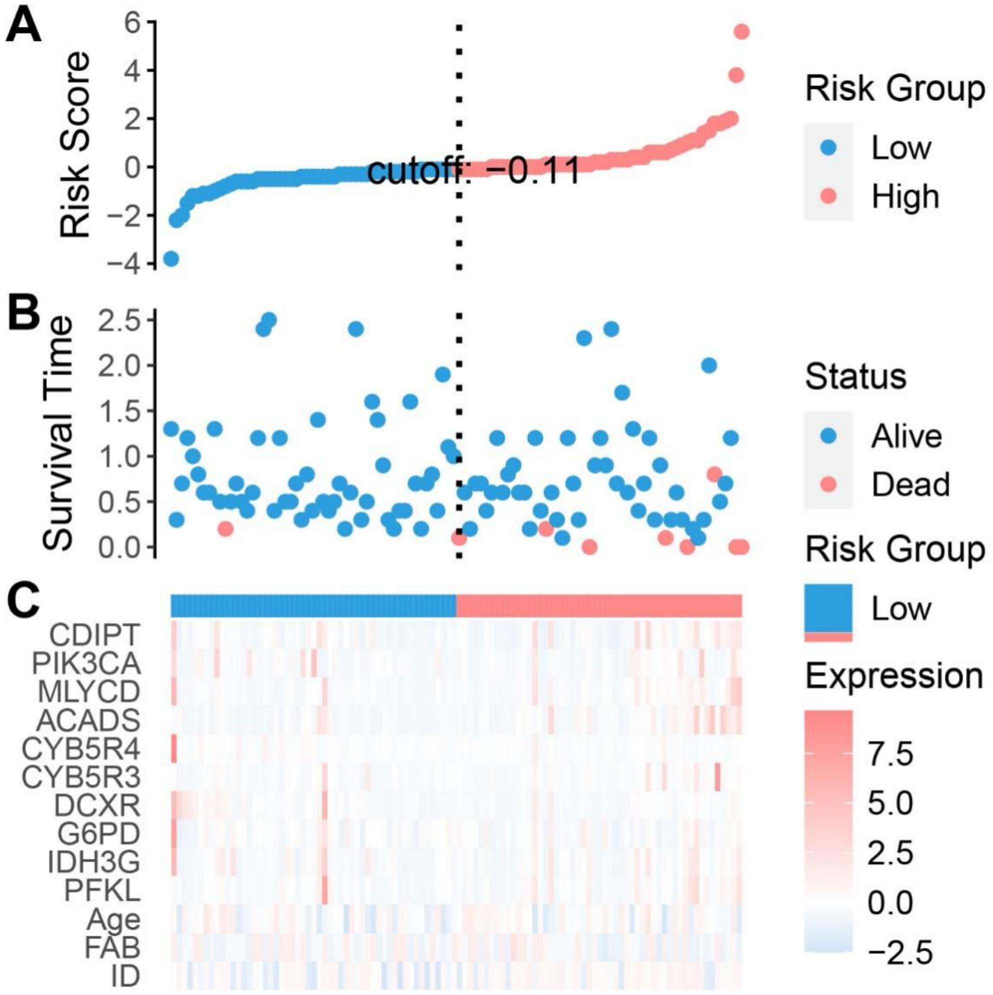

Supplementary Figure S12. Prognostic analysis of the CRA-related gene signature in the test set (Our cohort).

(A) Risk Groups based on patients' risk scores. (B) Patients' survival status along with their risk scores. (C) The expressions of the 10 model CRGs in the high- and low-risk groups.

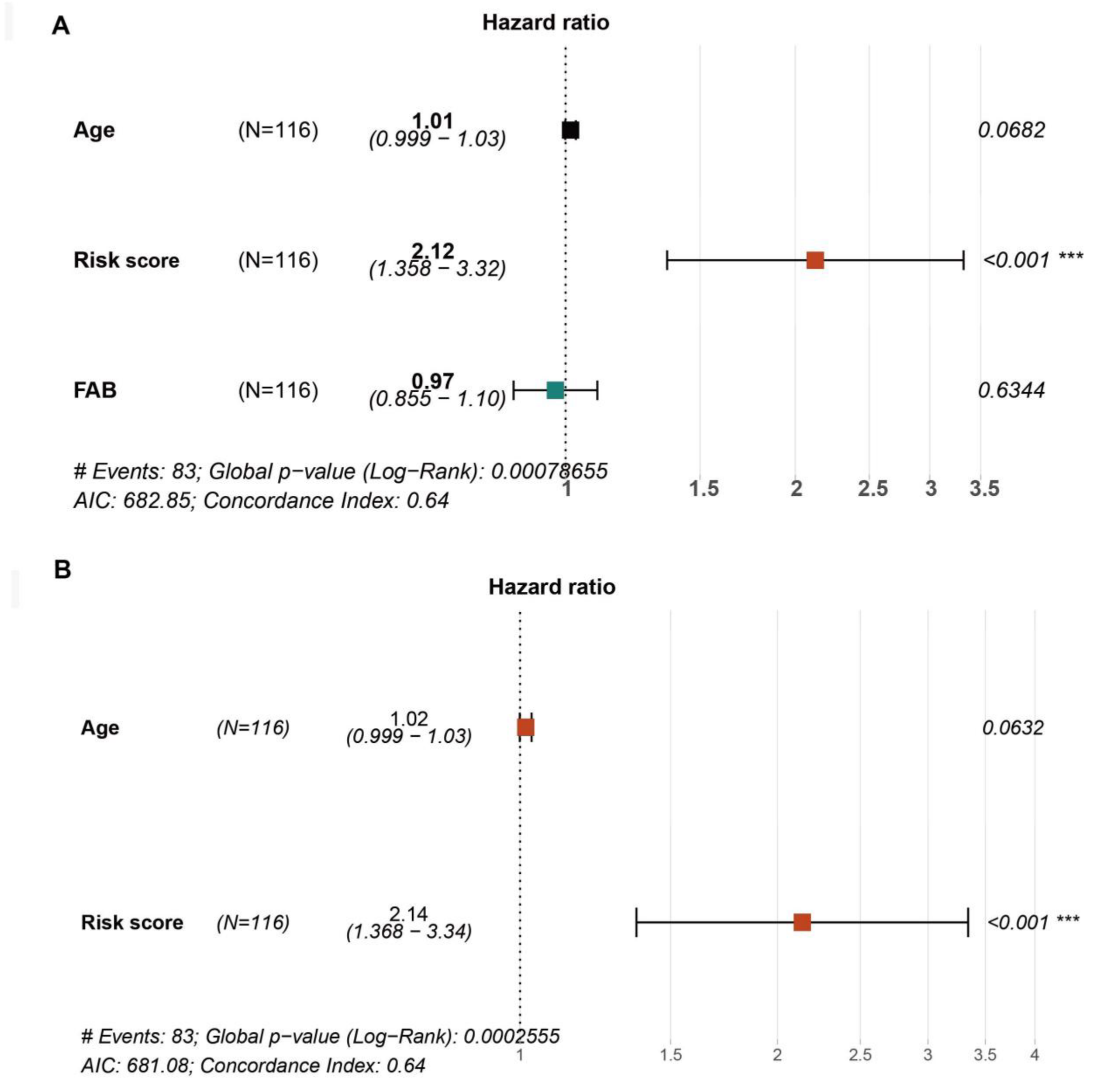

Supplementary Figure S13. Cox regression analysis of the risk scores and clinical parameters (GSE37642, GPL570)

(A) Univariate Cox regression analysis (B) Multivariate Cox regression analysis

*p < 0.05; **p < 0.01; ***p < 0.001; ns, not significant.

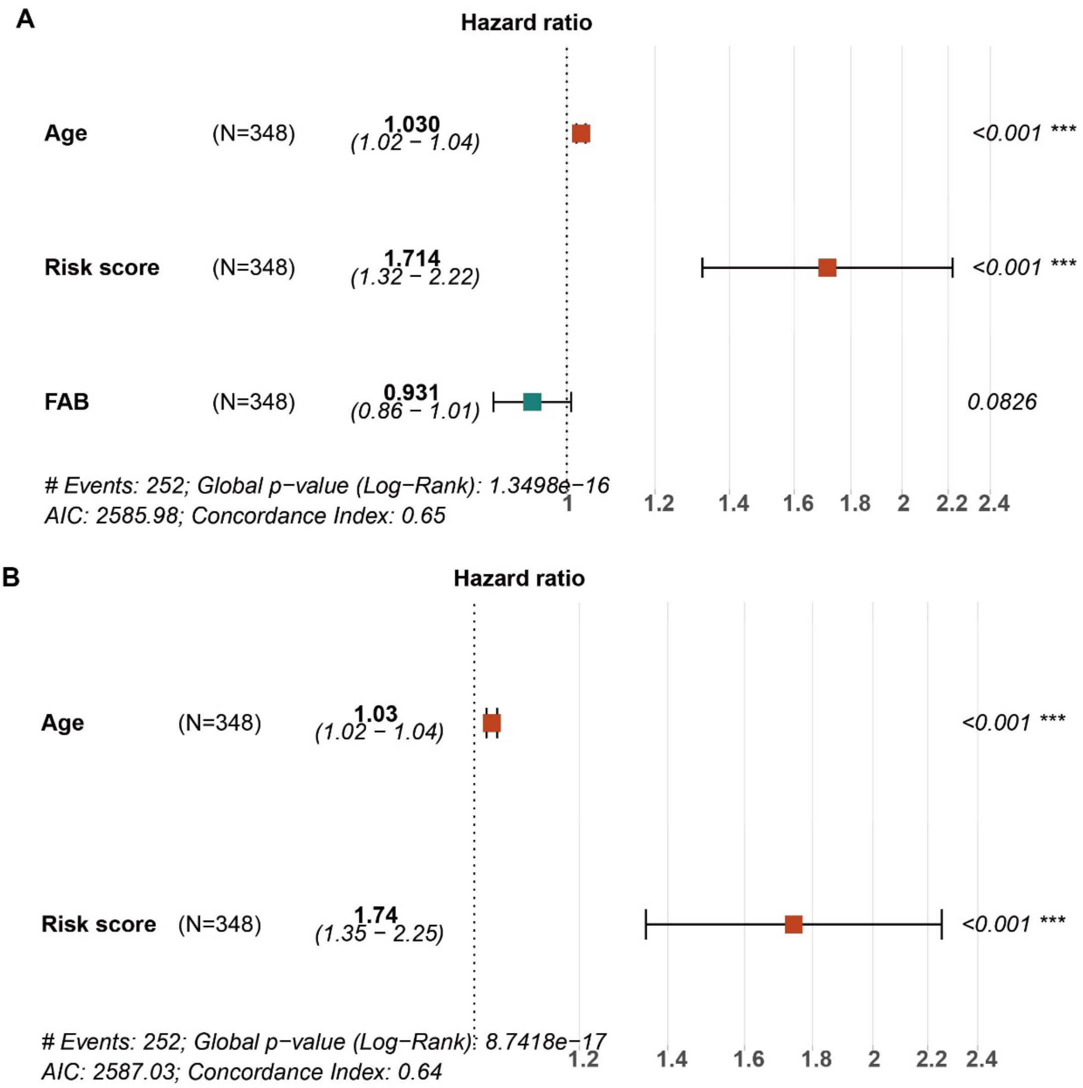

Supplementary Figure S14. Cox regression analysis of the risk scores and clinical parameters (GSE37642, GPL96)

(A) Univariate Cox regression analysis (B) Multivariate Cox regression analysis

*p < 0.05; **p < 0.01; ***p < 0.001; ns, not significant.

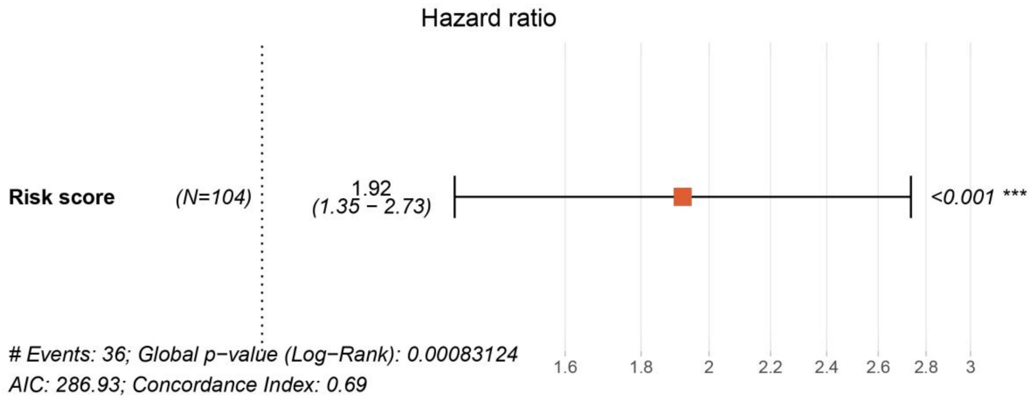

Supplementary Figure S15. Univariate Cox regression analysis of the risk scores and clinical parameters (GSE71014, GPL10558)

*p < 0.05; **p < 0.01; ***p < 0.001; ns, not significant.

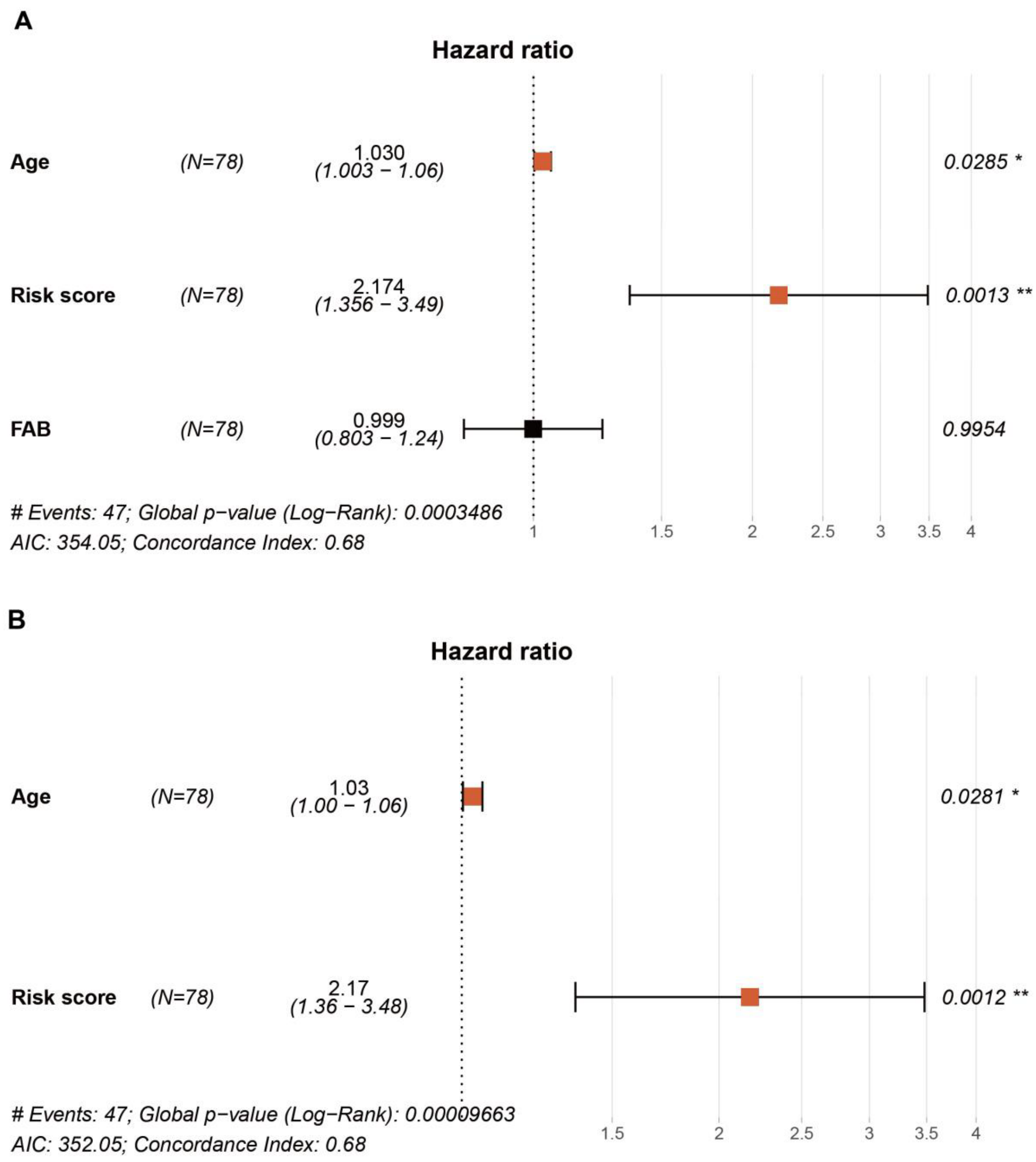

Supplementary Figure S16. Cox regression analysis of the risk scores and clinical parameters (GSE12417, GPL570)

(A) Univariate Cox regression analysis (B) Multivariate Cox regression analysis

*p < 0.05; **p < 0.01; ***p < 0.001; ns, not significant.

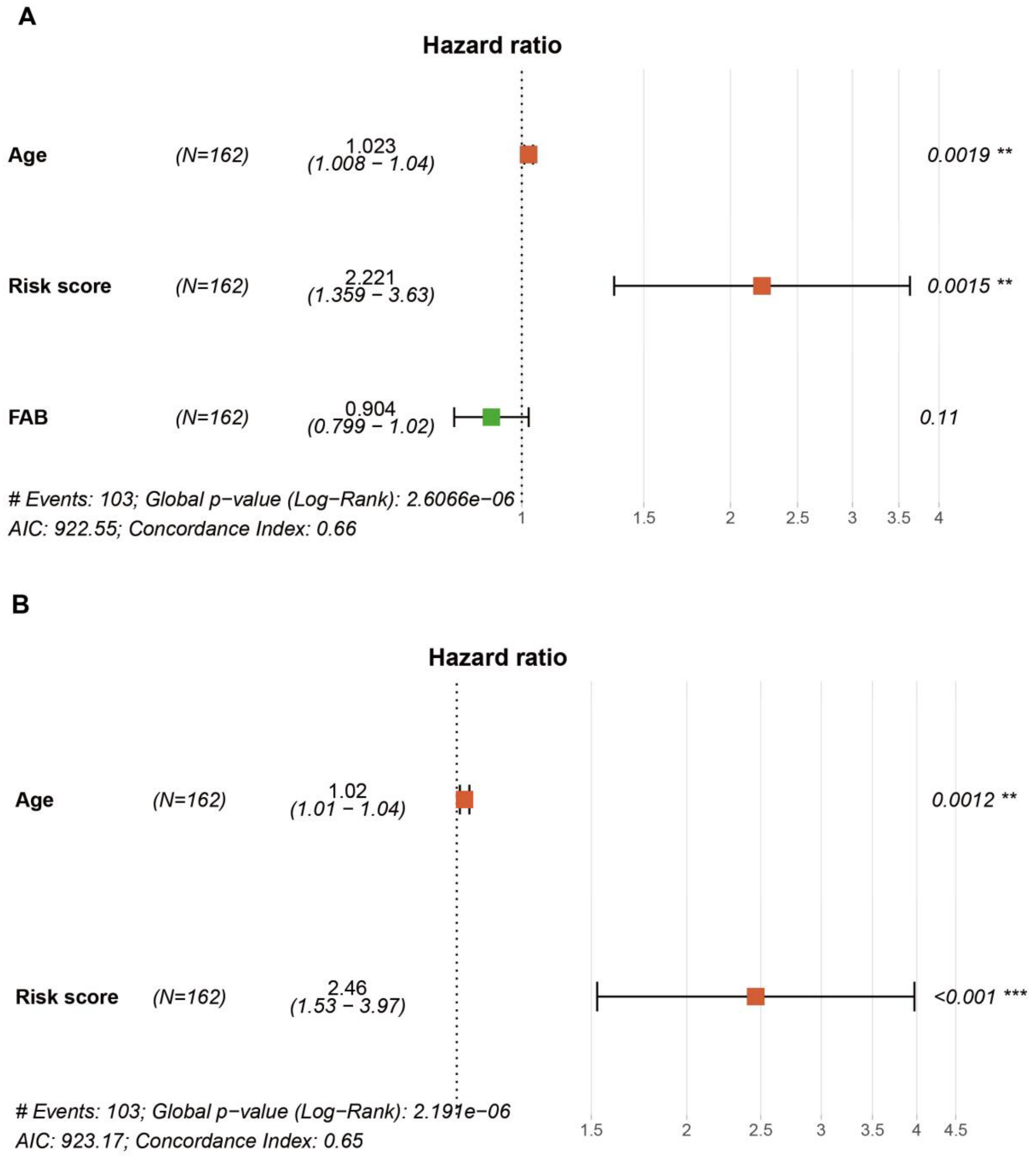

Supplementary Figure S17. Cox regression analysis of the risk scores and clinical parameters (GSE12417, GPL96)

(A) Univariate Cox regression analysis (B) Multivariate Cox regression analysis

*p < 0.05; **p < 0.01; ***p < 0.001; ns, not significant.

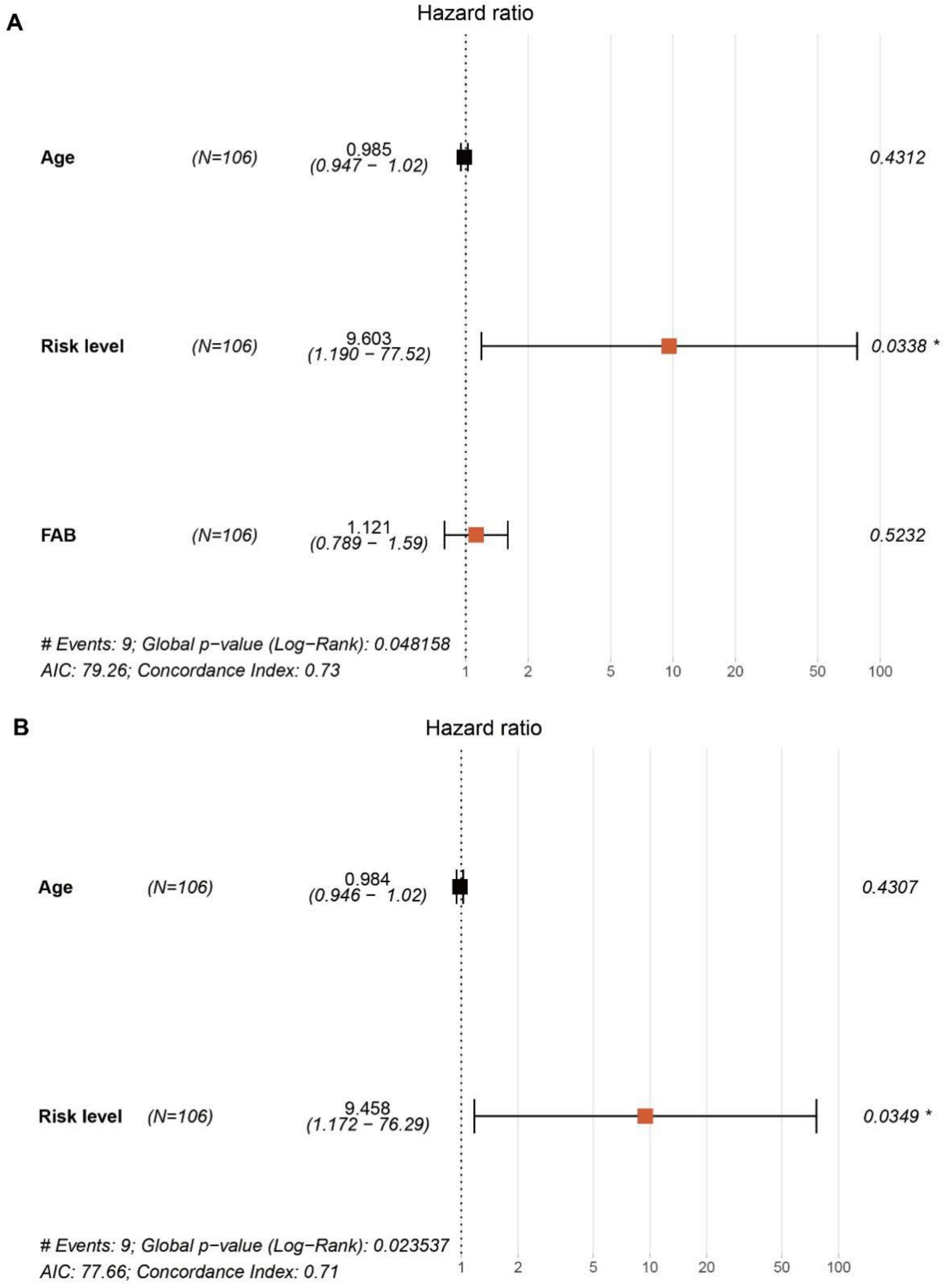

Supplementary Figure S18. Cox regression analysis of the risk scores and clinical parameters (Our cohort)

(A) Univariate Cox regression analysis (B) Multivariate Cox regression analysis

*p < 0.05; **p < 0.01; ***p < 0.001; ns, not significant.

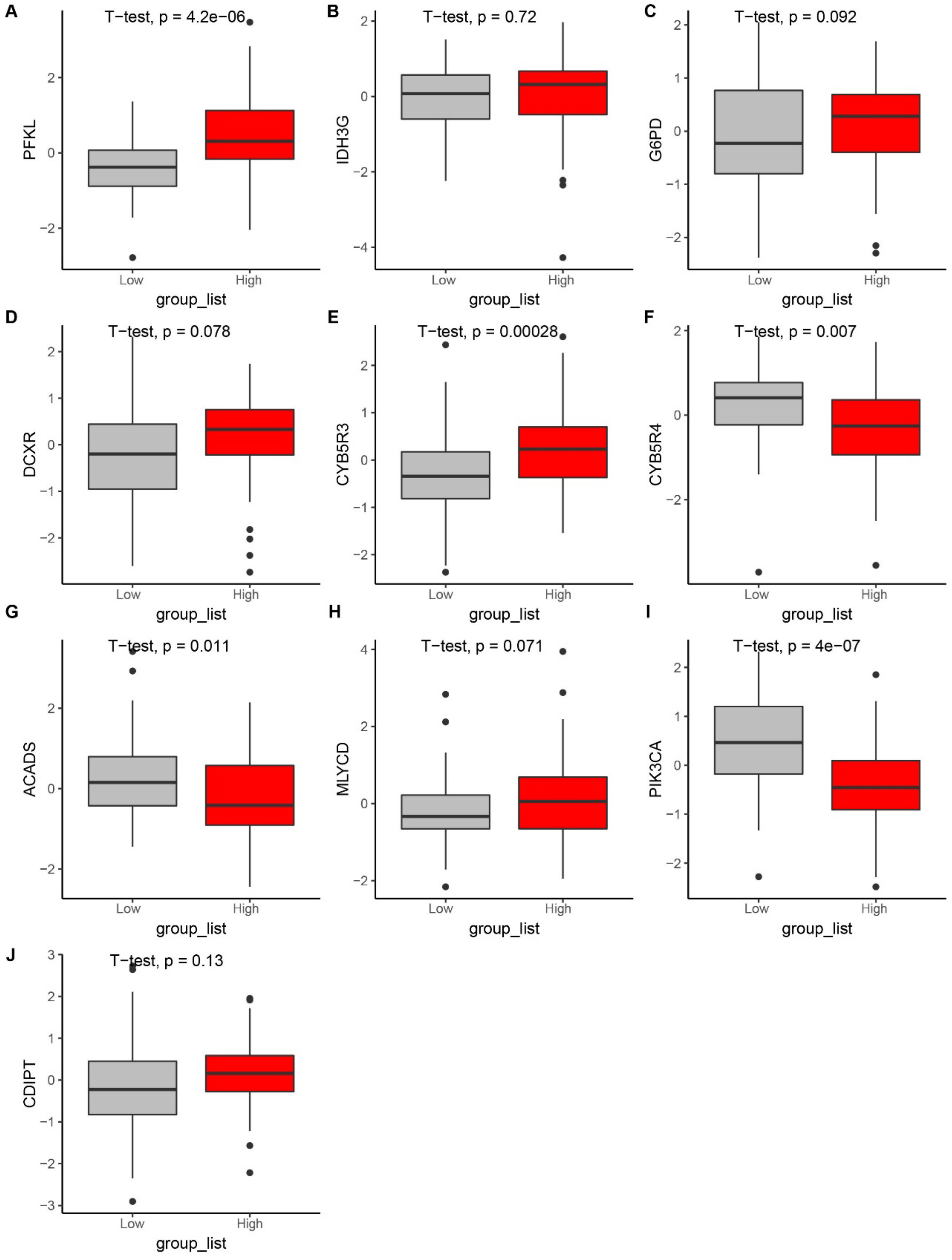

Supplementary Figure S19. Relative mRNA expression levels of 10 CRGs in high and low risk groups (GSE37642, GPL570)

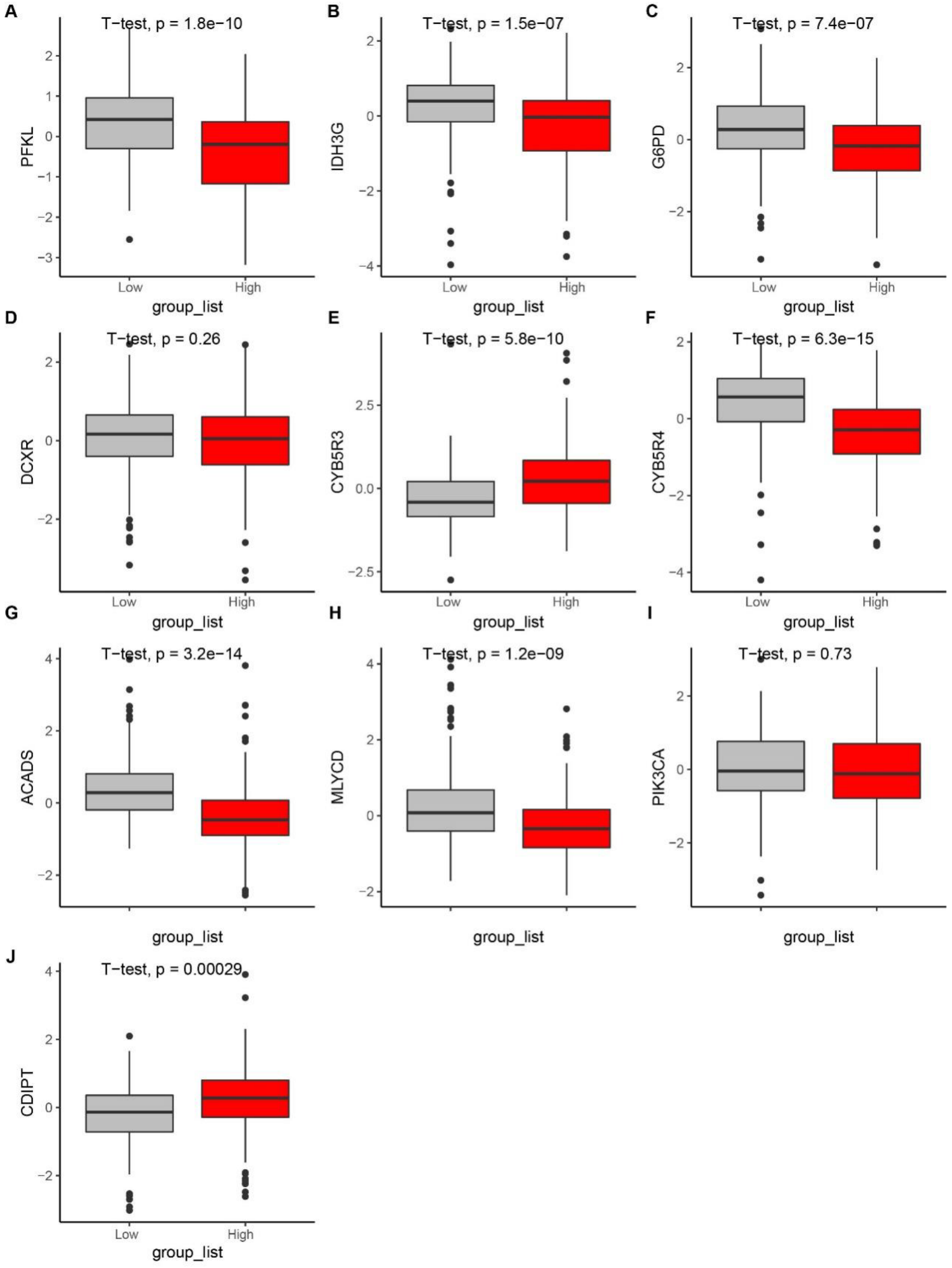

Supplementary Figure S20. Relative mRNA expression levels of 10 CRGs in high and low risk groups (GSE37642, GPL96)

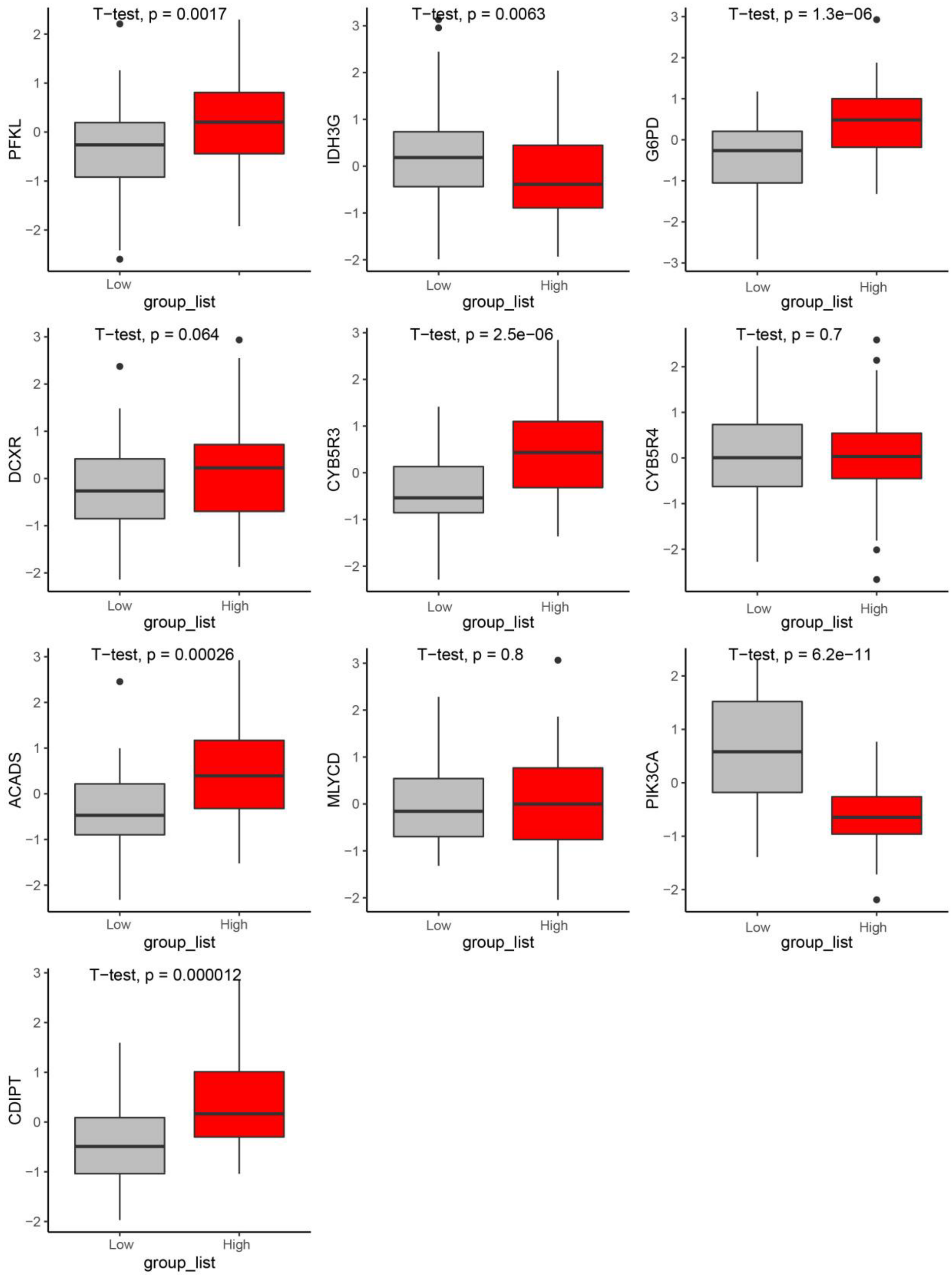

Supplementary Figure S21. Relative mRNA expression levels of 10 CRGs in high and low risk groups (GSE71014, GPL10558)

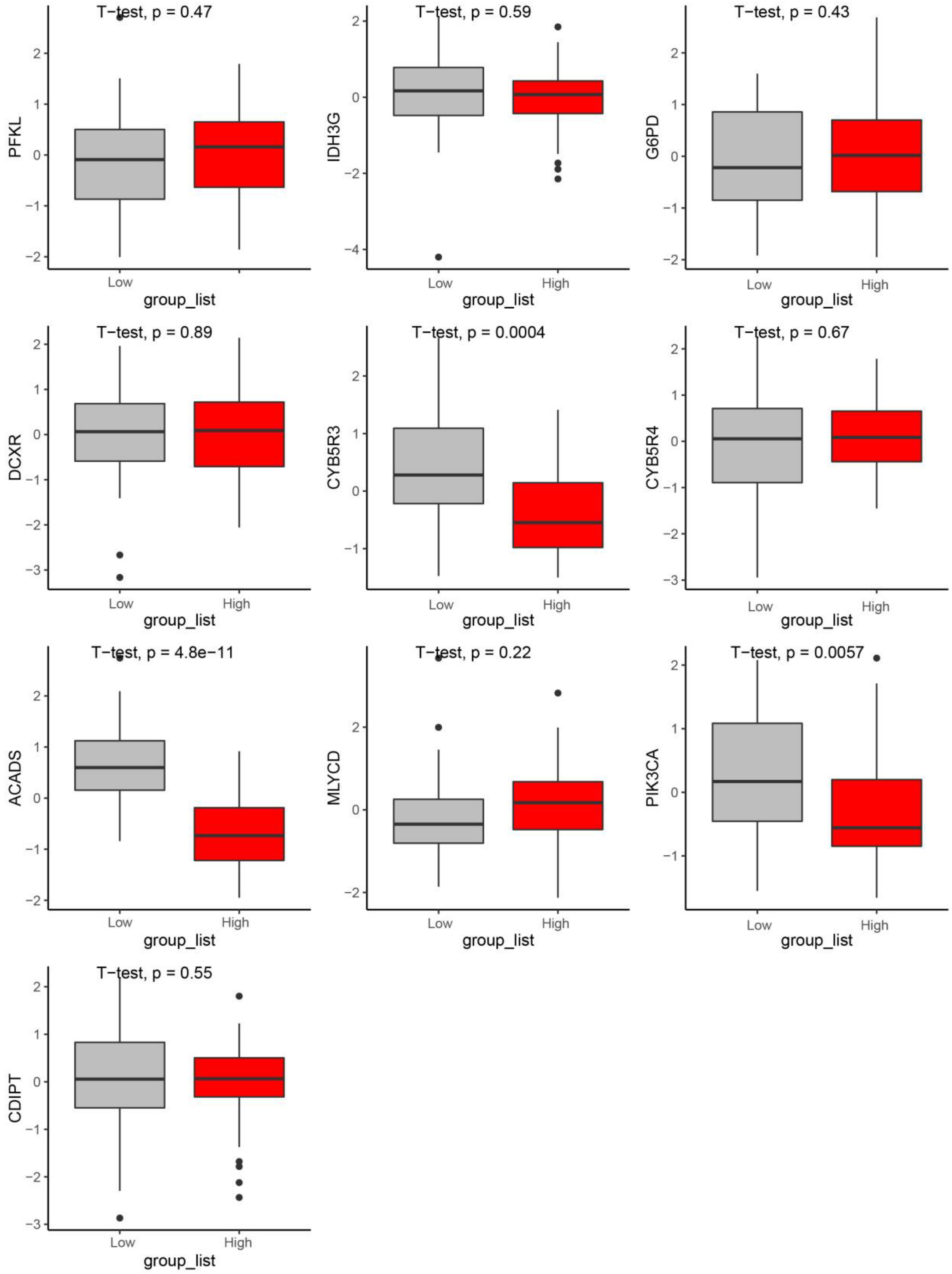

Supplementary Figure S22. Relative mRNA expression levels of 10 CRGs in high and low risk groups (GSE12417, GPL570)

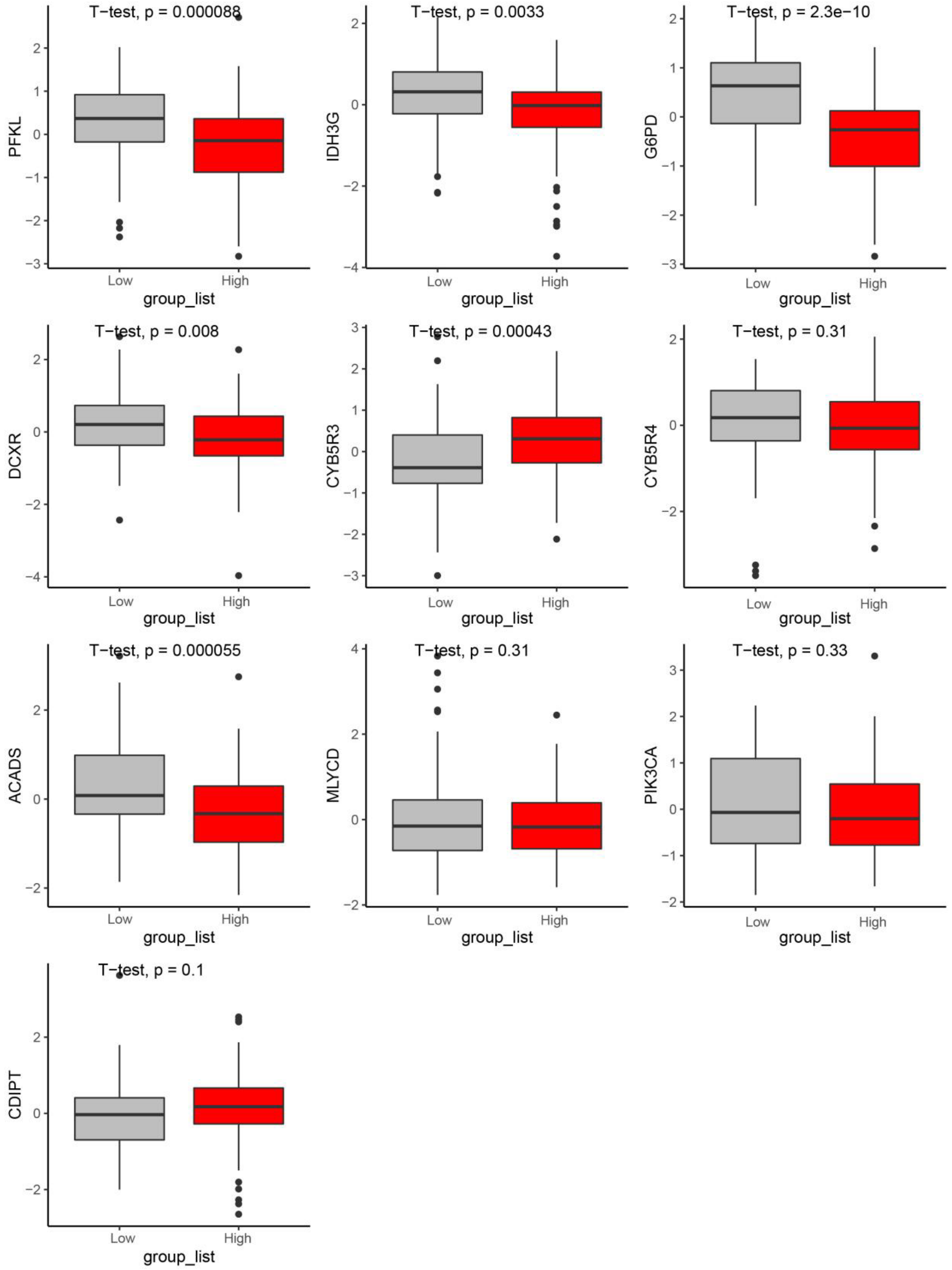

Supplementary Figure S23. Relative mRNA expression levels of 10 CRGs in high and low risk groups (GSE12417, GPL96)

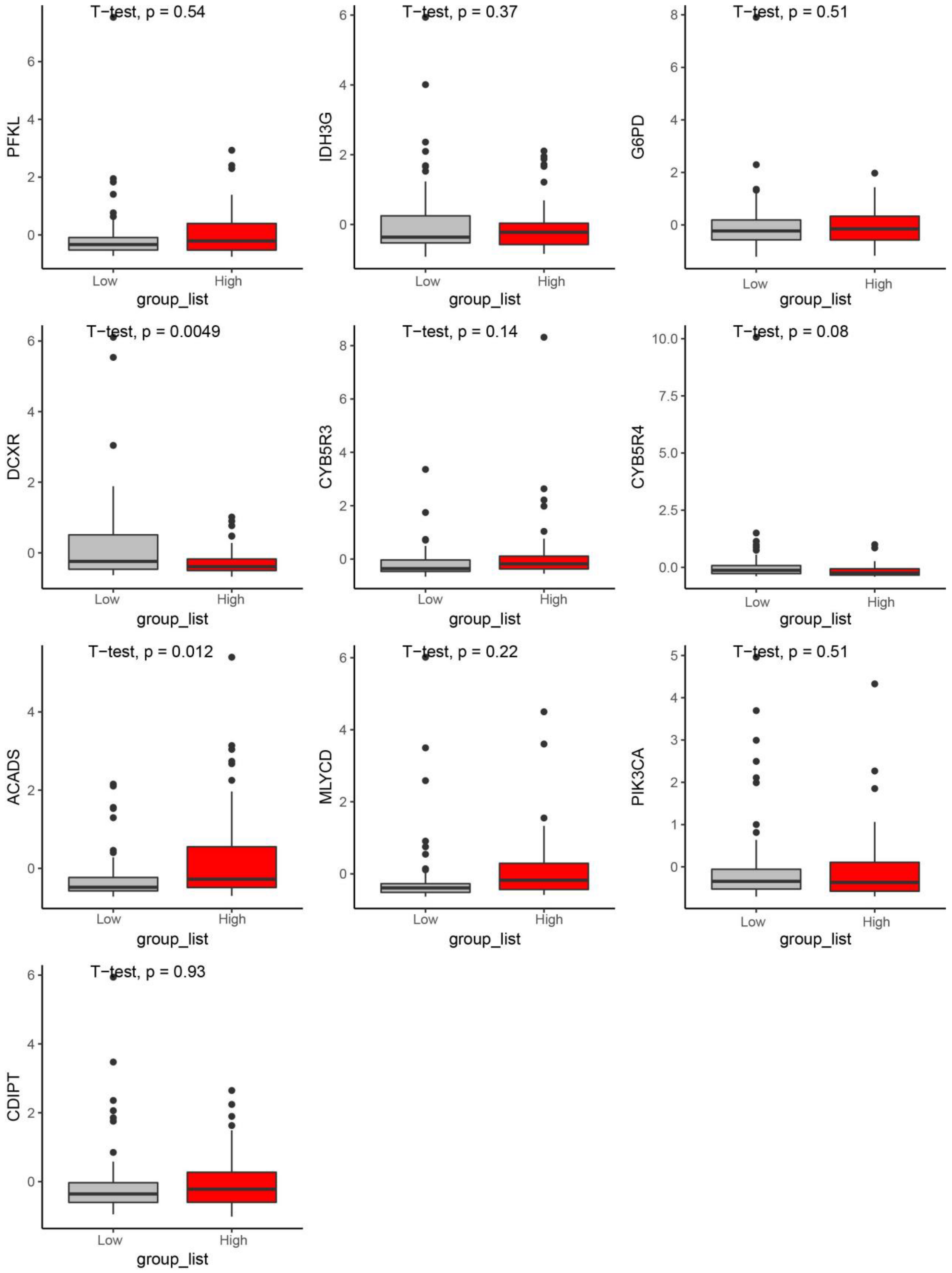

Supplementary Figure S24. Relative mRNA expression levels of 10 CRGs in high and low risk groups (Our cohort)

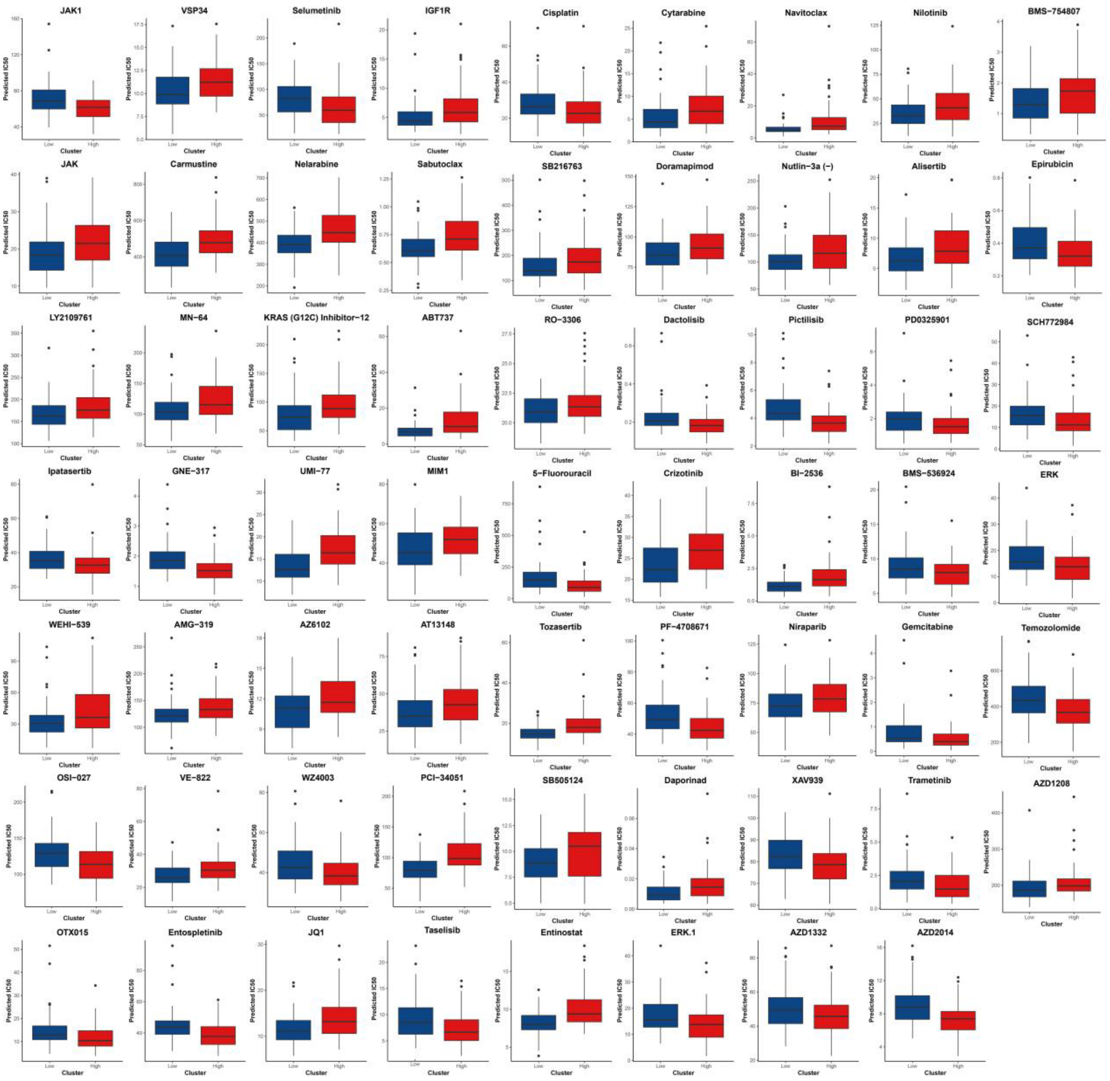

Supplementary Figure S25. Potential clinical chemotherapeutic response

IC50 indicates the half maximal inhibitory concentration.

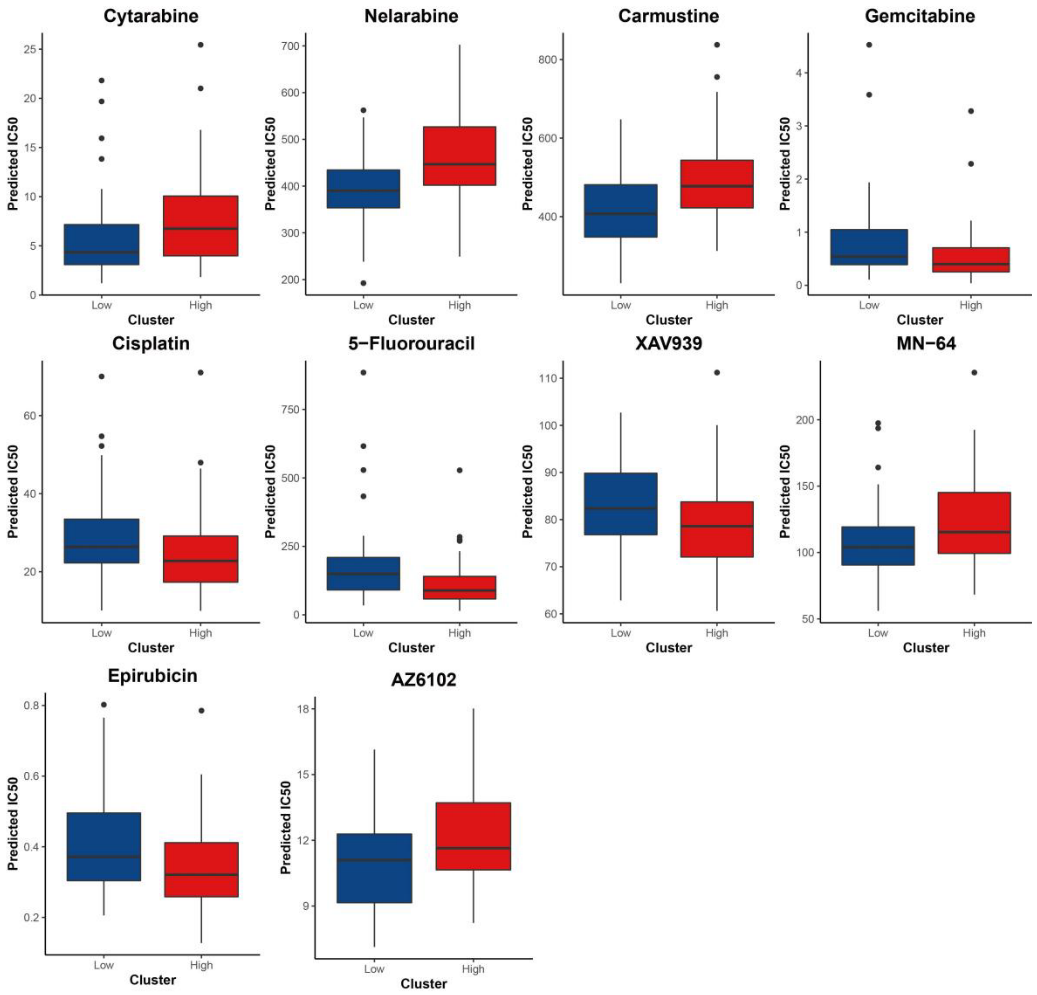

Supplementary Figure S26. Drugs of inhibiting DNA synthesis and inducing its damage

IC50 indicates the half maximal inhibitory concentration.

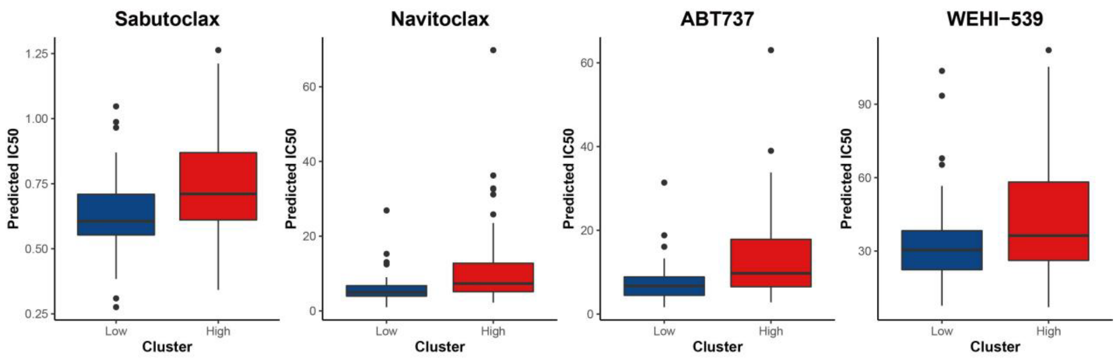

Supplementary Figure S27. Inhibitors of bcl2 family

IC50 indicates the half maximal inhibitory concentration.

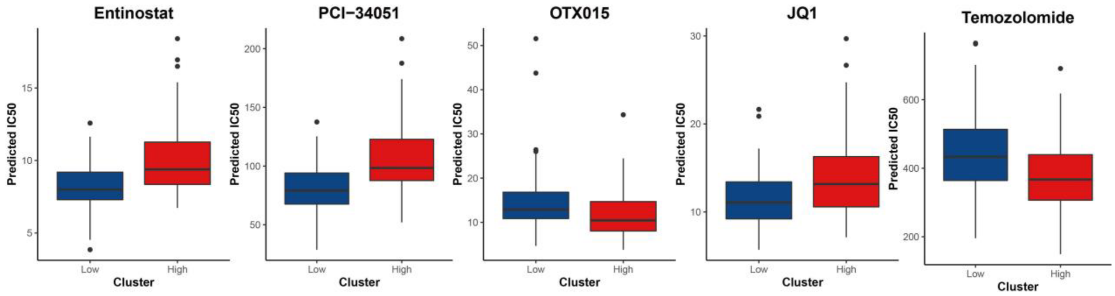

Supplementary Figure S28. Epigenetically Related Drugs

IC50 indicates the half maximal inhibitory concentration.

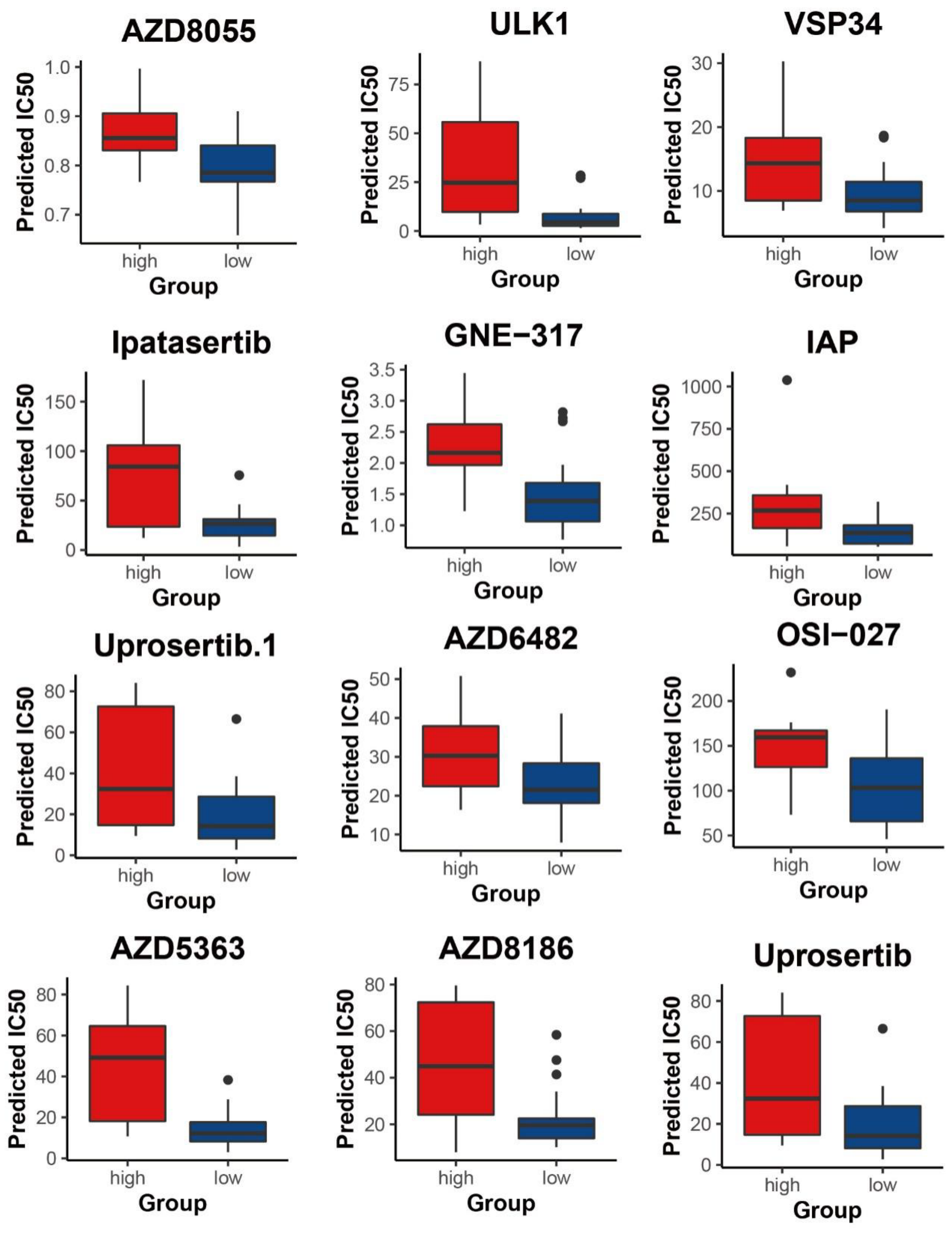

Supplementary Figure S29. Inhibitors of PI3K/AKT/mTOR signaling pathway

IC50 indicates the half maximal inhibitory concentration.
